# Supplementary material for: A multi-reservoir extruder for time-resolved serial protein crystallography and compound screening at X-ray free-electron lasers
Source: Nat Commun. 2023 Dec 2;14:7956. doi: 10.1038/s41467-023-43523-5 (PMC10693631; doi:10.1038/s41467-023-43523-5)
Supplement: Supplementary file 1 — Supplementary Information [file 41467_2023_43523_MOESM1_ESM.pdf]

Supplementary Materials

Supplementary Information: Detailed description of the design and operation of the multi-reservoir extruder.

Supplementary Material on SolQ2Br: Compound synthesis together with NMR spectra**.**

Supplementary Material on Sample Preparation: Additional information on sample preparation.

Crystallographic Table 1: Data processing Statistics.

Crystallographic Table 2: Data Refinement Statistics.

Supplementary Figure 1: Cross-sectional and oblique views of the multi-reservoir high viscosity extruder showing the rotational liquid feedthrough channels used to circulate the cooling liquid.

Supplementary Figure 2: The reservoir assembly.

Supplementary Figure 3: Sample interaction region of the multi-reservoir extruder.

Supplementary Figure 4: Detailed view of the plunger assembly.

Supplementary Figure 5: Graphical user interface for the multi-reservoir extruder.

Supplementary Figure 6: KR2 extrapolated maps, $2F_{extra}–F_c$ at 19% activation level for 1μs time delay.

Supplementary Figure 7: Comparison between drug cocktail soaking and colchicine soaking.

Supplementary Figure 8: "Data analysis titration" – site 1 ligand occupancy maps obtained from different amount of indexed frames.

Supplementary Figure 9: "Data analysis titration" – site 2 ligand occupancy maps obtained from different amount of indexed frames.

Supplementary Table 1: Sample extrusion speeds obtained from fast camera recordings compared with the extrusion speeds predicted from the flow equation.

Supplementary Information

General

The multi-reservoir extruder includes the main device as well as an additional electronics box containing several components. The main device measures 330 × 250 × 190 mm. The attached electronics box, which must also sit inside the Prime chamber, measures 150 × 150 × 125 mm. A Power Brick LV-IMS (Delta Tau) is used to control the device. Cables from the device and the electronics box are connected to a 24 V power supply and the Power Brick via a vacuum compatible flange on the Prime chamber. The device and its accessories are controlled through Experimental Physics and Industrial Control System (EPICS) via a graphical user interface.

As stated in the main text, the device comprises three key modules: the reservoir holder, the plunger assembly and the sample interaction region (**Figure 1A**). The device is loaded with up to nine sample-containing reservoir assemblies. These are all described in detail below.

The reservoir assemblies

The sample reservoir assemblies comprise several parts with the key part being the sample reservoir which contains the sample. The additional parts are used to fix the reservoir assemblies within the multi-reservoir extruder and to accommodate extrusion of the sample via a piston actuated mechanically using a stepper motor.

The sample reservoirs (**Figure 1B** and **Supplementary Figure 2**) are analogous to previously published designs[1-3]. The sample channel of the sample reservoirs has an inner diameter of 2.2 mm and a total length of 39 mm. This is sealed at one end by compressing a 2.5 mm diameter Teflon ball into the reservoir channel using a custom tool (Supplementary Figure 2). The sample can then be injected from a syringe into the reservoir channel by way of an adaptor to a final volume of approximately 130 µL. The sample reservoir is then sealed with a polyimide coated fused silica capillary needle (75 µm ID, 363 µm OD) Polymicro Technologies™, Molex®), fitted with a 12 mm PEEK tubing sleeve (0.15 in ID, 1/16 in OD) and a LT-100X LiteTouch® Ferrule (IDEX) assembly. This capillary needle assembly is fixed in place onto the reservoir channel using a nut, tightened to 2.0 Nm using a torque wrench.

The additional parts of the sample reservoir assembly include a plunger, a brass sheath, a spring and a bayonet sleeve. The plunger sits inside the brass sheath and the spring and bayonet sleeve are loaded onto the outside of this brass sheath. The brass sheath can then be screwed into the sample reservoir via a threaded connection bringing these additional parts together with the sample reservoir to form the complete sample reservoir assembly. Once assembled, the head of the plunger is able to contact the Teflon ball within the reservoir channel at the back of the

sample. The tail of the plunger has a flat surface which is accessible to the plunger assembly through an opening in the brass sheath. It is this contact which permits sample extrusion. The function of the spring and bayonet sleeve is to hold the sample reservoir assembly in place within the reservoir holder drum.

The reservoir holder

The reservoir holder can hold up to nine reservoirs and is responsible for selecting the desired reservoir, readying it to be delivered to the sample interaction region. A belt drive mechanism powers the reservoir holder drum. The drum moves unidirectionally and is actuated by a stepper motor (ST4118M906-B, Nanotec) equipped with an incremental encoder on its motor shaft (NOE2-05-K14 (5MM), Nanotec). A forked photoelectric sensor (PM-F45, Panasonic) is used to identify a reference position and align the reservoir holder drum. This is achieved via detection of a reference marker mounted on the moving part of the drum.

The reservoir holder drum was 3D-printed from CL 20ES stainless steel (Kyburz Feinmechanik AG) in order to facilitate the incorporation of liquid feedthrough channels surrounding each of the nine reservoir positions (Supplementary Figure 1). The liquid feed-through channels can be connected to a circulating chiller (Julabo FN25 EH) in order to stabilise the temperature of the reservoir holder drum at 20ºC for peak stability of LCP samples. It was possible to reach sample reservoir temperatures of 12ºC to 45ºC inclusive, as measured using a thermocouple attached to a sample reservoir mounted in the reservoir holder (Supplementary Figure 2A).

The sample interaction region

The sample interaction region sits on a stationary mount and comprises: 1) a fibre optical sensor, to detect the incoming sample capillary during sample selection and loading, 2) a guiding aperture to direct and align the incoming capillary needle, 3) a gas aperture mount, to house the ceramic gas aperture and to connect to the incoming sheath gas supply, 4) a YAG crystal in the plane of the jet, for beam diagnostic purposes, 5) a tip cleaner, to remove built up debris and sample from the capillary tip and 6) a 'catcher', connected to a vacuum pump, to collect extruded sample and stabilise the sample jet (Supplementary Figure 3).

To position the tip of the capillary at the sample interaction region, the reservoir holder containing loaded reservoirs and the plunger assembly are translated together by way of a linear stage. This linear stage has built in limit switches and an incremental encoder (MTM-120, Owis). In short, after the selected reservoir has been rotated into position by the reservoir holder assembly, the linear stage moves it forward towards the sample interaction region. This motion threads the capillary needle into the sample interaction region, ready for extrusion to take place. It is also responsible for retracting the needle and reservoir once the sample reservoir is empty.

After the guiding aperture, the capillary is driven through a small hole with an approximate diameter of 430 μm which forms the 'back' of the gas aperture mount. This seals the 'back' of

the capillary in the gas aperture mount sufficiently well so that He gas flow is preferentially delivered through the larger opening of the ceramic gas aperture around the extruded sample.

In order to accommodate variable capillary needle lengths – as these are produced manually and can have millimetres of variation in length – a fibre optical sensor (LL3-DZ02, GLL170-P332, Sick) is mounted in front of the guiding aperture at the entrance to the sample interaction region. As the capillary needle tip moves over the sensor, a reflection of the emitted light (wavelength, 632 nm) occurs and, once detected, provides a homing reference for the motion of the capillary needle towards the sample interaction region. The light from the sensor is turned off once this procedure has taken place to avoid light contamination of the sample. The distance between this sensor and the opening of the ceramic gas aperture (ref, Arizona State University. Centre for Biological Physics) is constant and during these experiments was calibrated as 26.5 mm. The reservoir and capillary needle could then be driven this distance automatically. This positioned the tip of the capillary needle in a reproducible but slightly retracted position with respect to the ceramic gas aperture opening. At this point, manual positioning of the capillary needle using the linear stage in fine, 50-100 µm steps could be carried out. This allowed for accurate and reproducible control of the position of the capillary needle and thus, viscous jet, with respect to the concentric gas flow from the ceramic aperture. This position could also be optimised during extrusion of the sample to flexibly permit maximum jet stability.

A detachable tip cleaner fitted with a disposable sponge was magnetically mounted below the sample interaction region onto a linear piezo-stage (SLC-1720, Smaract). The capillary tip and ceramic gas aperture could thus be cleaned without the need to break the vacuum environment of the Prime chamber during operation. Cleaning of the tip was also performed once a sample reservoir was depleted to ensure that residual sample present on the capillary tip was not deposited inside the stationary ceramic gas aperture upon retraction of the capillary needle during the automated sample reservoir exchange sequence.

A ‘catcher’ was magnetically mounted downstream of the capillary needle on a linear piezo-stage (SLC-1720, Smaract) and was connected to a vacuum pump. The remote motion of the catcher permitted on-the-fly stabilisation and tuning of the sample jet stability. The catcher had a conical opening with diameter 9.3 mm feeding into a cylindrical body with diameter 19 mm and length 60 mm. Using a dry scroll vacuum pump (nXDS 10iC, New Edwards) fitted with a gas flow meter (Vögtlin), a vacuum was applied to the catcher. The catcher was fitted with a removable mesh filter and both the catcher and filter could be removed and cleaned during sample reservoir exchanges.

The plunger assembly

The plunger assembly is responsible for extruding the sample at a desired flow rate and velocity (Supplementary Figure 4). A stepper motor (SCA2018M0804-A, Nanotec) and gear box (GPLE22-2S-9, Nanotec) are connected via a belt drive to a plunger actuator. The motor drives this plunger actuator into the back of the plunger of a sample reservoir assembly. The plunger

is then pushed forward, extruding the sample from the sample reservoir through the capillary needle at a defined velocity.

During data collection, the plunger actuator generally moves at velocities in the low µm/s range. However, a second, faster speed (500 µm/s) is used during sample exchange to reduce the downtime between samples. The issue is that when the plunger actuator moves the plunger with high speed into the sample, a fast pressure build up can result in sample loss and/or potential damage to the capillary needle or ceramic gas aperture. To avoid this rapid pressure change, a load cell (Subminiature load cell 8413, Burster) monitors the applied force of the plunger actuator and as soon as the force exceeds a defined value, the plunger motor is stopped and the plunger actuator retracts 500 µm from its current position. When the user is ready to begin sample extrusion for data collection, the motor can be restarted at the desired reduced velocity. The load cell and its in-line amplifier provide a 0 - 10 V signal, which correspond to 0 - 200 N. The signal is processed by a threshold relay, which can be adjusted by a potentiometer.

Positive and negative limit switches (IFFM 08P37A6/L, Baumer) sit at each end of the 40 mm travel range of the plunger actuator. As well as limiting the motion of the plunger actuator, these permit an accurate readback of the amount of sample remaining at any given point during sample extrusion. The limit switches, combined with the continuous readback of the force exerted on the load cell during sample extrusion mean that blockages of the capillary needle can be easily distinguished from the sample reservoir being empty.

Temperature control tests

Temperature control tests of the multiinjector assembly were carried outside the Alvra Prime chamber at atmospheric pressure and ambient temperature (23ºC). Acting as a thermocouple, DT-421 Series Silicon Diode was used to measure the temperature of an empty reservoir with respect to the demand temperature of the circulating cooling fluid within the reservoir holder drum. The thermocouple was connected to a single reservoir via a vacuum feedthrough. The thermocpule was connected to LakeShore Temperature control unit (Model 331) which was used to automatically log the temperature. The reservoir was then placed inside one of the reservoir holder drum positions analogous to how it would be during sample extrusion.

The temperature of the circulating fluid within the reservoir holder drum was set using a Julabo F25-EH circulator filled with Thermal-G (Julabo). The temperature of the thermocouple attached to the reservoir was recorded manually while setting the circulator temperature between 5ºC and 45ºC in 5 degree increments. The temperature of the thermocouple at each set point was left to stabilize such that the temperature did not change over a timescale of 10 minutes or longer.

A standard curve of the demand temperature set on the circulator versus the readback from the thermocouple attached to the reservoir was calculated (Figure 1C). When increasing the

temperature in 5 degree increments, the circulator took approximately 7 minutes to stabilize at the demand temperature (to 0.25ºC precision). The corresponding change of the thermocouple took up to 20 minutes (to 1ºC precision). Temperatures below 5ºC were not attempted due a risk of ice or condensation on the circulator lines and multiinjector assembly. Temperatures above 45ºC were avoided due to the operating temperature limits of the motors of the multiinjector assembly.

References

1. Weierstall, U. *et al.* Lipidic cubic phase injector facilitates membrane protein serial femtosecond crystallography. *Nat. Commun.* **5**, 3309 (2014).
2. Botha, S. *et al.* Room-temperature serial crystallography at synchrotron X-ray sources using slowly flowing free-standing high-viscosity microstreams. *Acta Cryst.* **D71**, 387–397 (2015).
3. Shimazu, Y. *et al.* High-viscosity sample-injection device for serial femtosecond crystallography at atmospheric pressure. *J. Appl. Crystallogr.* **52**, 1280–1288 (2019).

Supplementary Material on SolQ2Br

Synthesis of solQ2Br

Building block sources, solvent grades, synthetic conditions, characterisation and purification procedures, and instrumentation, were as previously described [https://doi.org/10.1021/jacs.2c01020].

2-hydroxyphenyl methanesulfonate (1):

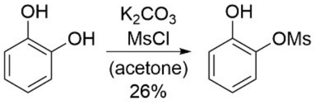

Commercially available catechol (1.0 g, 9.0 mmol, 1.0 eq.) was dissolved in dry acetone (30 mL). Finely ground K_2CO_3 (2.5 g, 18 mmol, 2.0 eq.) and methanesulfonyl chloride (MsCl, 0.78 mL, 10 mmol, 1.1 eq.) were added and the reaction mixture was heated to reflux, stirring at 50°C for 3 h until full consumption of starting material. After cooling to ambient temperature, K_2CO_3 was filtered off and the filtrate concentrated under reduced pressure. The obtained residue was taken up in dichloromethane (DCM, 20 mL), acidified to pH 1 with aqueous HCl (2 M, 200 mL) and extracted with DCM (4 x 50 mL). The combined organic phases were washed with brine (1 x 100 mL), dried over Na_2SO_4, filtered and concentrated *in vacuo* to give the crude product as a yellow oil. Flash column chromatography on silica (hexane:EtOAc, 5:1 → 1:1) afforded the known mono-mesylated catechol (0.44 g, 2.3 mmol, 26%) that matched literature data [https://doi.org/10.1002/chir.20055].

^{1}H NMR (400 MHz, $CDCl_3$, 300 K): δ [ppm] = 7.24 (dd, *J* = 8.2, 1.6 Hz, 1H), 7.13 (ddd, *J* = 8.2, 7.4, 1.6 Hz, 1H), 7.06 (br. s, 1H), 6.98 (dd, *J* = 8.2, 1.6 Hz, 1H), 6.87 (ddd, *J* = 8.2, 7.4, 1.6 Hz, 1H), 3.19 (s, 3H).

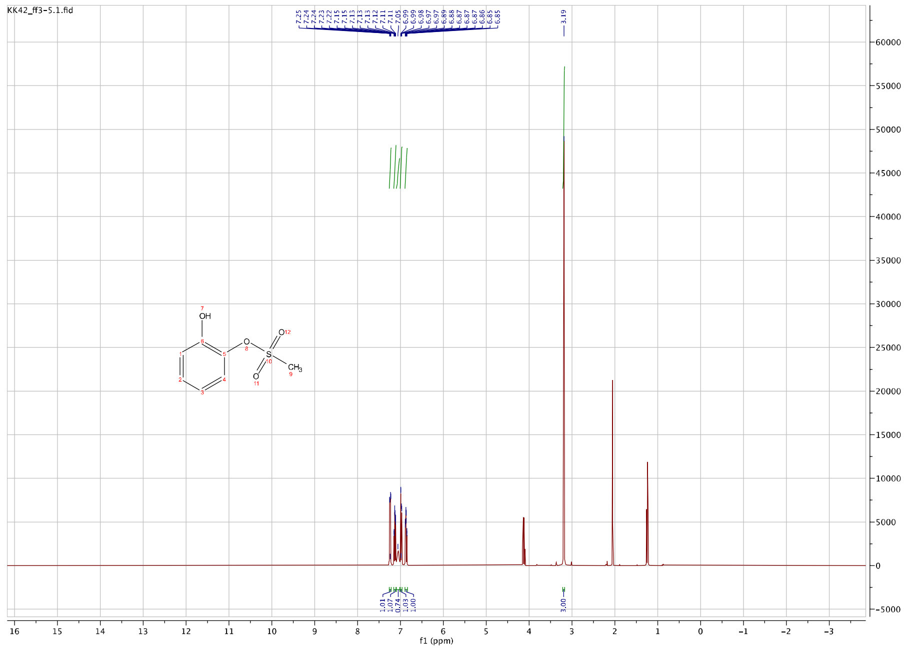

4-(2-(benzyloxy)ethyl)-3,5-dimethoxyaniline (2):

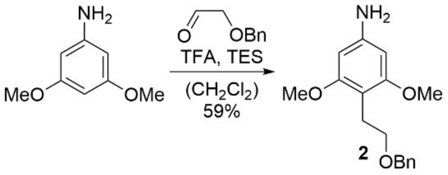

Analogously to the elegant *para*-alkylation procedure for electron rich anilines optimised by Augustine [https://doi.org/10.1055/s-2008-1078207], to a solution of commercially available 3,5-dimethoxyaniline (1.0 g, 6.7 mmol, 1.0 eq.) and benzyloxyacetaldehyde (1.0 g, 6.7 mmol, 1.0 eq.) in DCM (25 mL) was added trifluoracetic acid (TFA, 1.0 mL, 13 mmol, 2.0 eq.). The mixture was stirred at room temperature for 10 min before being cooled in an ice bath. To this cooled solution triethylsilane (1.8 mL, 11 mmol, 1.7 eq.) was added dropwise. The reaction mixture was allowed to warm to ambient temperature and left stirring for 3 h. The crude mixture was then diluted with KH_2PO_4/K_2HPO_4-buffer (50 mL) and extracted with DCM (4 x 50 mL). The combined organic phases were washed with brine (1 x 100 mL), dried over Na_2SO_4, filtered and concentrated *in vacuo*. Flash column chromatography on silica (hexane:EtOAc, 5:1 → 1:1 + 1% MeOH) gave aniline **2** as a beige solid (1.1 g, 3.8 mmol, 57%).

TLC (hexane:EtOAc 1:1): R_f = 0.40 [UV, CAM]. **^{1}H NMR** (400 MHz, $CDCl_3$, 300 K): δ[ppm] = 7.38 – 7.20 (m, 5H), 5.89 (s, 2H), 4.55 (s, 2H), 3.72 (s, 6H), 3.54 (dd, J = 8.8, 7.0 Hz, 2H), 2.94 (dd, J = 8.8, 7.0 Hz, 2H). **HRMS-ESI (m/z):** calc. for $[C_{17}H_{22}NO_3]^+$ ($[M+H]^+$): 288.15942; found: 288.15941.

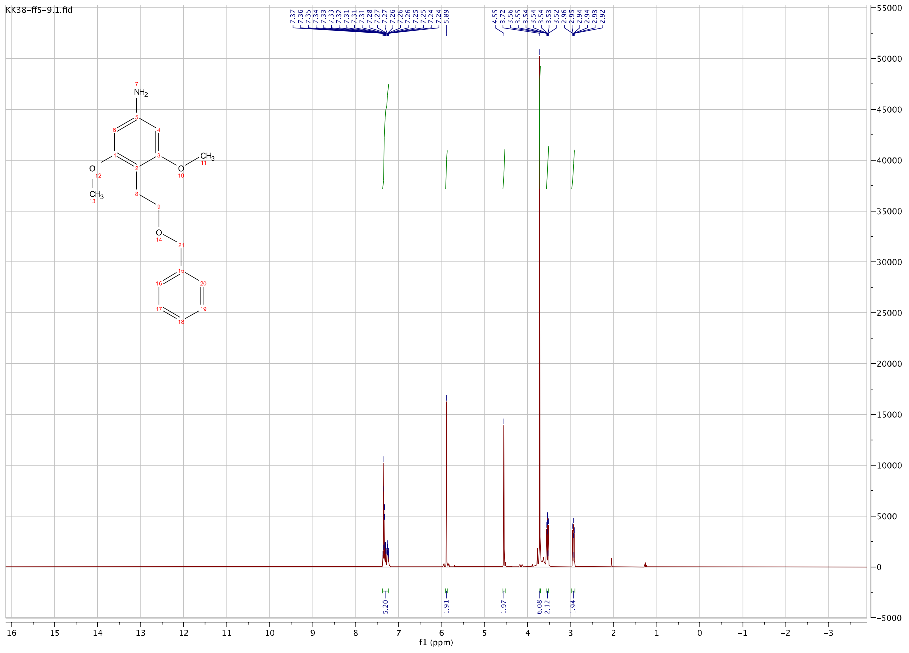

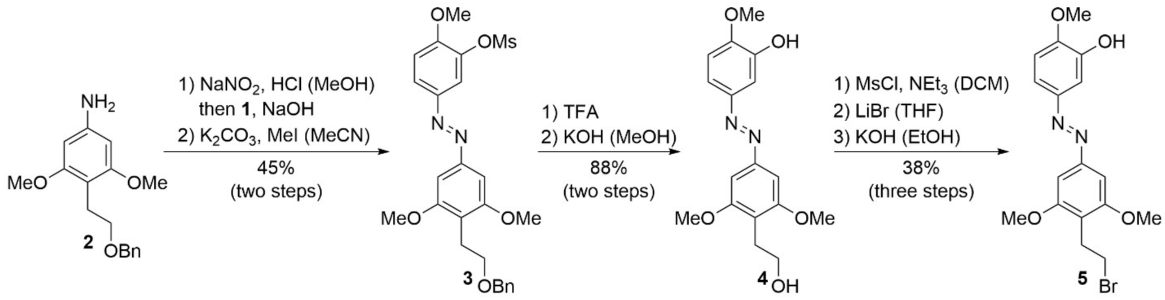

5-((4-(2-(benzyloxy)ethyl)-3,5-dimethoxyphenyl)diazenyl)-2-methoxyphenyl methanesulfonate (3):

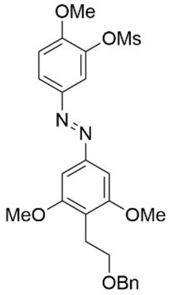

To the aniline **2** (0.56 g, 1.95 mmol, 1.0 eq.) were added methanol (MeOH, 5 mL) and conc. HCl (1 mL), while cooling the mixture in an ice bath. An aqueous solution of $NaNO_2$ (2.3 M, 0.80 mL, 1.95 mmol, 1.2 eq.) was added dropwise and the mixture was left stirring for 15 min in the cold. This diazonium solution was then transferred dropwise into a well-stirred cold solution of phenol **1** (0.44 g, 2.34 mmol, 1.2 eq.) in aqueous NaOH (2.0 M, 1.2 mL, 1.2 eq.). During and after the addition, the pH was maintained at 9<pH<12 by adjusting when necessary. The mixture was left stirring overnight until complete consumption. MeOH was removed under reduced pressure and the crude aqueous mixture was extracted with ethyl acetate (EtOAc, 3 x 20 mL). The combined organic layers were then washed with water (2 x 20 mL), brine (2 x 20 mL), dried over Na_2SO_4 and filtered. The crude *para*-phenolic intermediate was concentrated and used without further purification.

To a solution of the crude intermediate in acetonitrile (MeCN, 5 mL) were added an excess of finely ground K_2CO_3 and methyl iodide (0.15 mL, 2.41 mmol, 1.2 eq.). The reaction mixture was heated to 80°C for 1h until full conversion was observed via TLC. The crude mixture was then diluted with water (30 mL) and extracted with EtOAc (3 x 15 mL). The combined organic phases were washed with water (2 x 10 mL), brine (1 x 10 mL), dried over Na_2SO_4 and filtered. The crude was concentrated *in vacuo* and purified by flash column chromatography on silica (hexane:EtOAc, 5:1 → 1:1) to yield the azobenzene **3** as an orange solid (0.44 g, 0.88 mmol, 45%).

TLC (hexane:EtOAc 1:1): R_f = 0.47 [UV, CAM]. **^{1}H NMR** (400 MHz, $CDCl_3$, 300 K): δ [ppm] = 7.95 – 7.92 (m, 2H), 7.36 – 7.24 (m, 5H), 7.15 (s, 2H), 7.13 (d, J = 8.0 Hz, 1H), 4.57 (s, 2H), 3.98 (s, 3H), 3.90 (s, 6H), 3.64 dd, J = 8.3, 7.1 Hz, 2H), 3.24 (s, 2H), 3.11 (dd, J = 8.3, 7.1 Hz, 2H). **^{13}C NMR** (101 MHz, $CDCl_3$, 300 K): δ [ppm] = 158.95 (s, 2C), 153.64 (s, 1C), 152.08 (s, 1C), 146.58 (s, 1C), 138.88 (s, 1C), 138.81 (s, 1C), 128.33 (s, 2C), 127.65 (s, 2C), 127.42 (s, 1C), 125.14 (s, 1C), 118.45 (s, 1C), 117.31 (s, 1C), 112.48 (s, 1C), 98.81 (s, 2C), 72.56 (s, 1C), 68.89 (s, 1C), 56.45 (s, 1C), 55.92 (s,

2C), 38.60 (s, 1C), 24.04 (s, 1C). **HRMS-EI (m/z):** calc. for $[C_{25}H_{28}N_2O_7S]^+$ ($[M+H]^+$): 500.16117; found: 500.1612.

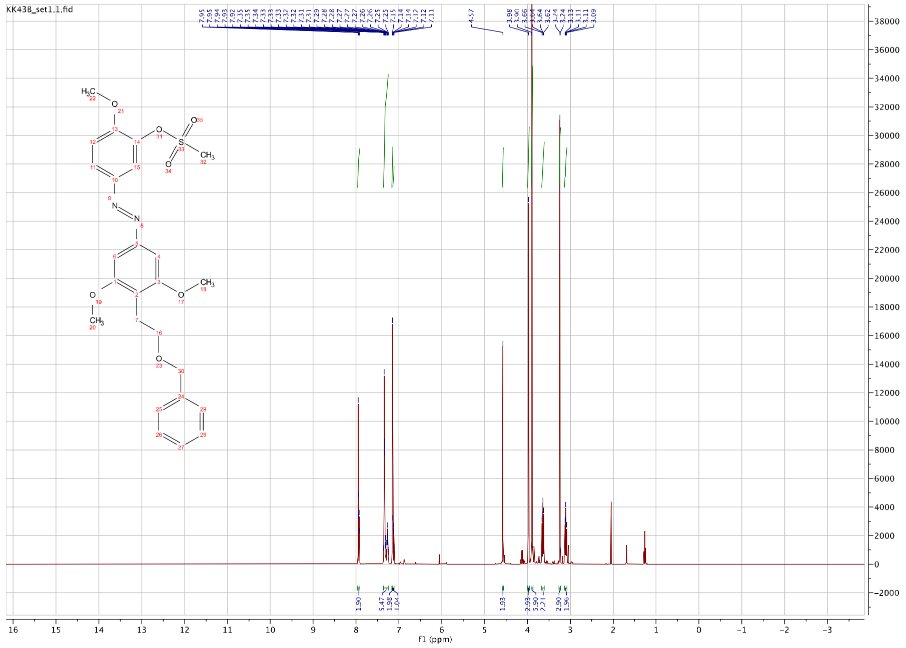

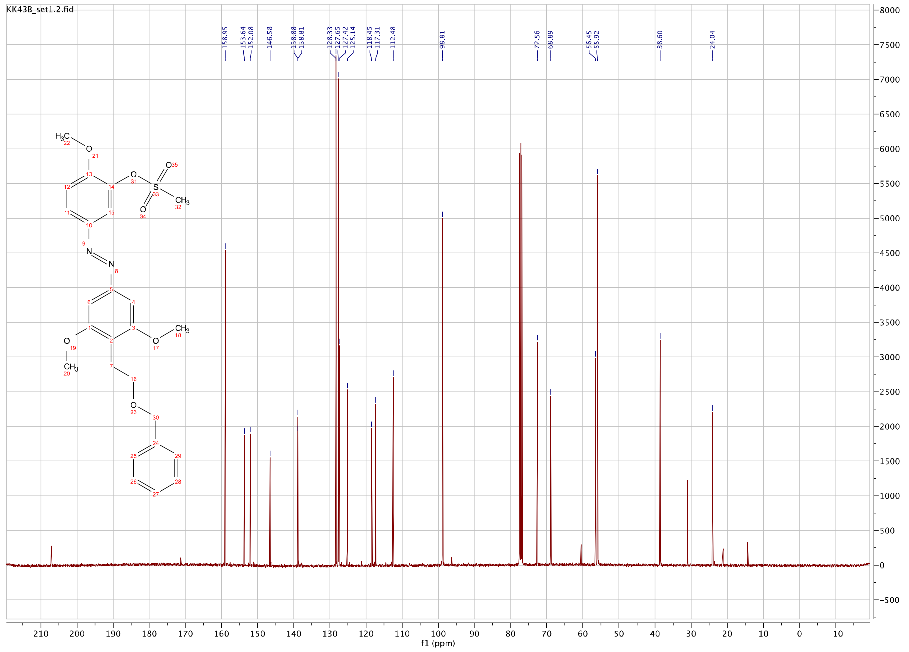

5-((4-(2-hydroxyethyl)-3,5-dimethoxyphenyl)diazenyl)-2-methoxyphenol (4):

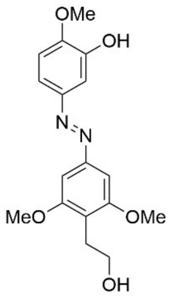

The azobenzene **3** (400 mg, 0.8 mmol, 1.0 eq.) was dissolved in neat trifluoroacetic acid (5 mL) and heated to 50°C. Reaction progress was checked via TLC and the mixture stirred at 50°C overnight until complete consumption of the starting material. The crude mixture was then concentrated under reduced pressure and redissolved in MeOH (3 mL). Solid KOH was added in excess and the reaction mixture quickly heated to reflux using a heat gun until full hydrolysis of the trifluoroacetic acid ester was observed via LC-MS. The crude mixture was acidified using aqueous HCl (2 M, 20 mL) and extracted with EtOAc (3 x 10 mL). The combined organic phases were washed with water (2 x 10 mL), brine (1 x 10 mL), dried over Na_2SO_4, filtered and concentrated under reduced pressure. Purification by flash column chromatography on silica (hexane:EtOAc, 2.5:1 → 1:1 + 1% MeOH) afforded the azobenzene **4** as an orange solid (232 mg, 0.7 mmol, 88%).

^{1}H NMR (400 MHz, $CDCl_3$, 300 K): δ [ppm] = 7.55 (dd, *J* = 8.5, 2.3 Hz, 1H), 7.53 (d, *J* = 2.3 Hz, 1H), 7.16 (s, 2H), 6.98 (d, *J* = 8.5 Hz, 1H), 3.98 (s, 3H), 3.92 (s, 6H), 3.80 (t, *J* = 6.5 Hz, 2H), 3.03 (t, *J* = 6.5 Hz, 2H). **^{13}C NMR** (101 MHz, $CDCl_3$, 300 K): δ [ppm] = 158.86 (s, 2C), 152.53 (s, 1C), 149.44 (s, 1C), 147.48 (s, 1C), 146.32 (s, 1C), 119.31 (s, 1C), 118.09 (s, 1C), 110.17 (s, 1C), 106.19 (s, 1C), 98.80 (s, 2C), 62.77 (s, 1C), 56.28 (s, 1C), 56.03 (s, 2C), 27.01 (s, 1C). **HRMS-ESI (m/z):** calc. for $[C_{17}H_{21}N_2O_5]^+$ ($[M+H]^+$): 333.14450; found: 333.14409.

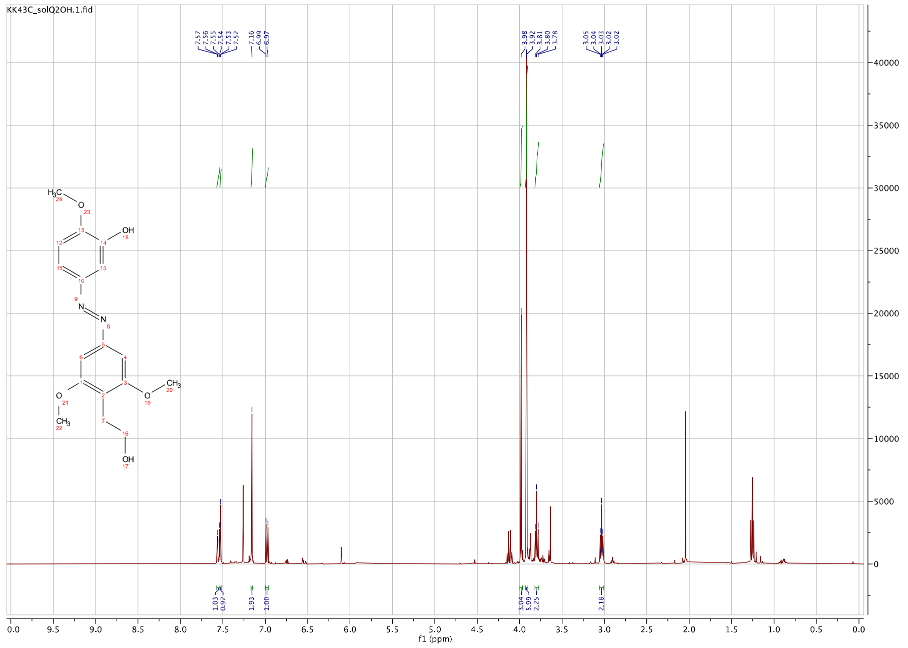

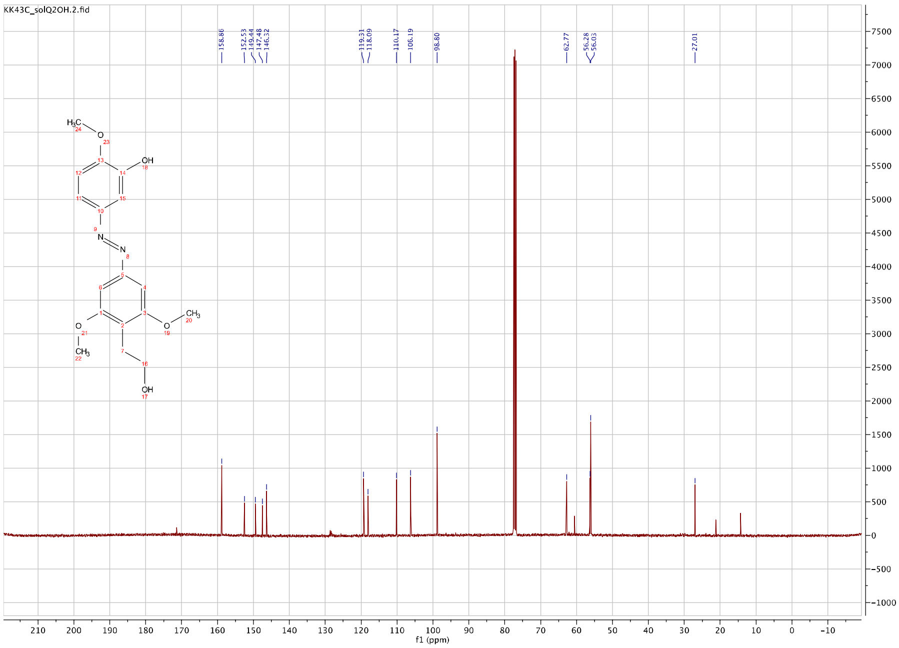

5-((4-(2-bromoethyl)-3,5-dimethoxyphenyl)diazenyl)-2-methoxyphenol (5, solQ2Br):

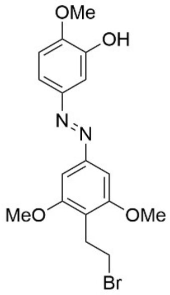

The conversion of **4** to **5** was carried out similar to literature procedures. [10.1002/adsc.201100736]

To the azobenzene **4** (27 mg, 0.08 mmol, 1.0 eq.) was added anhydrous DCM (2 mL) under an atmosphere of nitrogen while cooling the mixture in an ice bath. To this cooled solution triethylamine (30 □L, 0.22 mmol, 2.8 eq.) and MsCl (20 □L, 0.26 mmol, 3.3 eq) were added. The reaction mixture was then allowed to warm to ambient temperature and left stirring overnight until consumption of the starting material was observed by LC-MS. The crude mixture was diluted with water (10 mL) and extracted with DCM (3 x 5 mL). The combined organic phases were washed with water (2 x 5 mL), brine (1 x 5 mL), dried over Na_2SO_4, filtered and concentrated under reduced pressure.

To this crude bismesylated azobenzene in THF (3 mL) was added solid LiBr in excess. The reaction mixture was heated to 75°C for 24 h and reaction progress was monitored via LC-MS. Following completion of the reaction after additionally stirring at room temperature for 60 h, the mixture was diluted with water (10 mL) and extracted with EtOAc (3 x 5 mL). The combined organic layers were washed with water (2 x 5 mL), brine (1 x 5 mL), dried over Na_2SO_4 and filtered. The crude was concentrated *in vacuo* and used without further purification.

The crude was dissolved in 1% KOH in ethanol (2 mL). The reaction mixture was stirred at room temperature for 15 min and the amount of KOH was adjusted to 5% until full conversion was observed

via TLC. The mixture was then diluted with water (15 mL) and extracted with EtOAc (3 x 10 mL). The combined organic layers were washed with water (2 x 10 mL), brine (1 x 10 mL), dried over Na_2SO_4 and filtered. The crude was concentrated under reduced pressure and purified by column chromatography (hexane:EtOAc, 5:1 → 1:1) to yield **solQ2Br** (**5**) as an orange solid (12 mg, 0.03 mmol, 38%).

TLC (hexane:EtOAc 1:1): R_f = 0.59 [UV, CAM]. **^{1}H NMR** (400 MHz, $CDCl_3$, 300 K): δ [ppm] = 7.56 (dd, *J* = 8.4, 2.3 Hz, 1H), 7.53 (d, *J* = 2.3 Hz, 1H), 7.13 (s, 2H), 6.99 (d, *J* = 8.4 Hz, 1H), 5.72 (br. s, 1H), 3.99 (s, 3H), 3.92 (s, 6H), 3.48 (dd, *J* = 9.3, 7.1 Hz, 1H), 3.28 (dd, *J* = 9.3, 7.1 Hz, 1H). **^{13}C NMR** (101 MHz, $CDCl_3$, 300 K): δ [ppm] = 158.78 (s, 2C), 152.83 (s, 1C), 149.47 (s, 1C), 147.48 (s, 1C), 146.32 (s, 1C), 119.44 (s, 1C), 117.96 (s, 1C), 110.16 (s, 1C), 106.14 (s, 1C), 98.66 (s, 2C), 56.30 (s, 1C), 56.00 (s, 2C), 31.07 (s, 1C), 27.53 (s, 1C). **HRMS-ESI (m/z):** calc. for $[C_{17}H_{20}BrN_2O_4]^+$ ($[M+H]^+$): 395.06010; found: 395.06013.

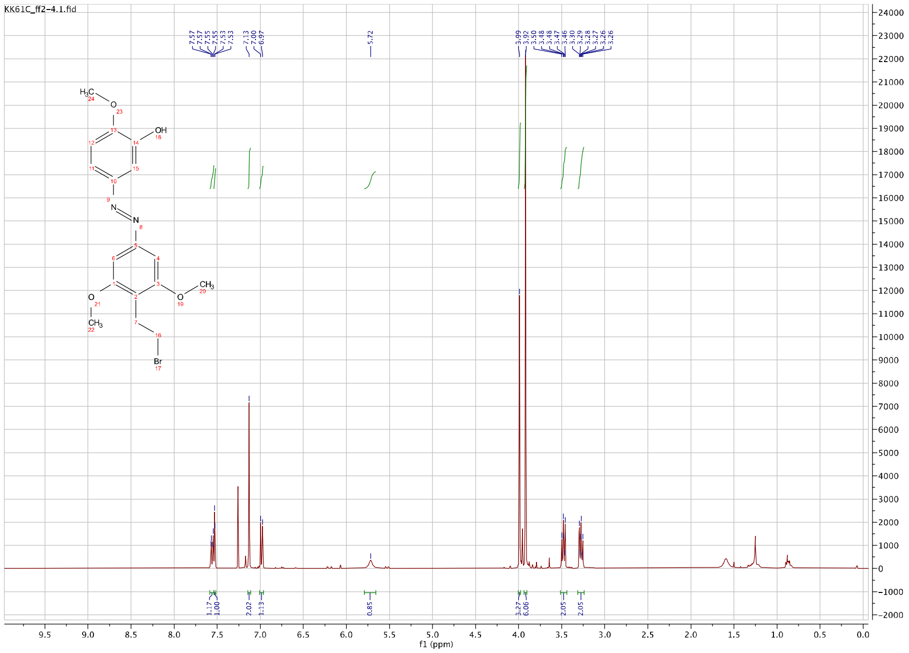

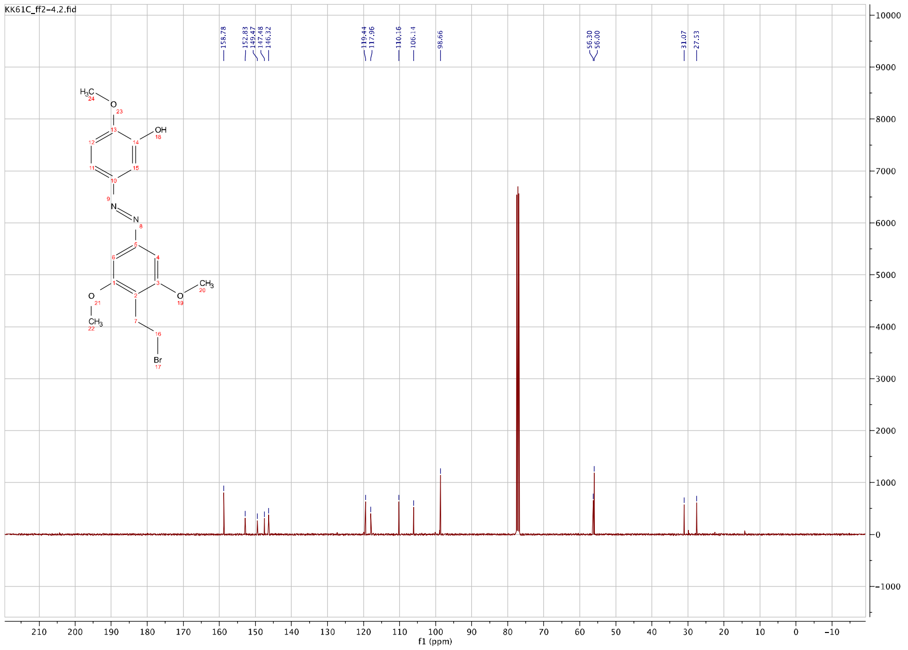
KK61C_ff2-4.2.fid
158.78
152.83
149.47
147.48
146.32
119.44
117.96
110.16
106.14
98.66
56.30
56.00
31.07
27.53
f1 (ppm)

Additional information on sample preparation

Sample preparation: tubulin-DARPin D1 (TD1) protein complex

Bovine brain tubulin was purchased from the Centro de Investigaciones Biológicas (Microtubule Stabilizing Agents Group), CSIC, Madrid, Spain. DARPin D1 was expressed and purified according to previous descriptions in ref. [1,2]. The large-scale production of high-resolution tubulin crystals is described in ref. [2,3].

The pET-derived vector PSTCm9 includes the gene for the artificial DARPin D1 as an N-terminal fusion with thioredoxin followed by a hexahistidine-tag and a thrombin cleavage site. For transformation, 0.5 µL of the DARPin D1-carrying plasmid was added to 10 µL of chemically competent *E.coli* BL21 (DE3) cells. After 10 minutes of incubation on ice, the cells were heat-shocked in a water-filled heating block at 42 °C for 15 seconds. After 10 minutes of incubation on ice, 100 mL of pre-heated SOC medium was added, and the cells were incubated for 2 hours at 37 °C and 180 rpm. In the following, the cells were plated on ampicillin-containing LB agar plates (100 µg/mL) and incubated overnight at 37 °C. The cells were suspended with 2 mL of pre-warmed LB medium and transferred to 200 mL LB medium supplemented with 200 µL of ampicillin solution (100 mg/mL). After the cells were incubated for 1 hour at 37 °C and 180 rpm shaking, 10 ml of cell solution were added to 1 L of pre-warmed LB medium supplemented with 1 mL of ampicillin solution (100 mg/mL) and incubated at 37 °C at 180 rpm. When the optical density OD_{600nm} reached a value of 0.7, the flasks were transferred to a different incubator with a set temperature of 18 °C. Expression of the plasmid coded DARPin D1 was induced 1 hour later by adding 1 mL of IPTG solution (1 M), and the cell suspension was incubated overnight while shaking at 180 rpm. The cells were harvested by centrifugation at 5,000 x g at 4 °C for 40 minutes. The supernatant was removed, cell pellets were combined in 50 mL Falcon tubes, flash-frozen in liquid nitrogen, and stored at - 80 °C. For a protein purification, cells were first thawed in a water bath at 25 °C and then resuspended with 150 mL of lysis buffer (50 mM Tris-HCl pH 7.5, 500 mM NaCl, 20 mM imidazole, 10% glycerol, 5 mM β-mercaptoethanol, DNAse I (25 µg/mL) and three protease inhibitor cocktail tablets (Roche). The cell walls were lysed on ice using an ultrasonic cell disrupter applying alterations of 2 seconds on and off pulses with 30% amplitude for 22 minutes. The lysate was centrifuged at 20,000 x g at 4 °C for 40 minutes, and the supernatant collected. The supernatant was filtered using a 0.45 µm pore size and applied to a Ni-NTA column on a fast protein liquid chromatography system (FPLC). After loading, the column was washed first with 30 column volumes of IMAC-binding buffer (50 mM Tris-HCl pH 7.5, 500 mM NaCl, 20 mM imidazole, 10% glycerol, 5 mM β-mercaptoethanol) and by additional 20 column volumes of IMAC-binding buffer combined with 4% IMAC-elution buffer (50 mM Tris-HCl pH 7.5, 500 mM NaCl, 500 mM imidazole, 10% glycerol, 5 mM β-mercaptoethanol). Protein elution was performed over 10 column volumes with 100% IMAC-elution buffer. The presence of DARPin D1 was confirmed by analyzing the eluted fractions on SDS-PAGE. The respective fractions were pooled together and diluted to 2 mg/mL with thrombin cleavage buffer (20 mM Tris-HCl pH 8.5, 150 mM NaCl, and 2.5 mM $CaCl_2$). After adding 2 units of thrombin, the solution was loaded into a pre-washed dialysis tubing (cutoff = 5 kDa) and dialyzed against 3 L of thrombin cleavage buffer at 4 °C for 72 hours. After cleavage, the solution was applied to a Ni-NTA column on a fast protein liquid chromatography system. After cleavage, most DARPin D1 could be collected from the flow through. Further fractions were collected separately after washing the column resin with first IMAC-binding buffer and then IMAC-elution buffer. All fractions with detectable UV absorbance at 280 nm were analyzed on SDS- PAGE. Pure fractions were collected and concentrated to a final volume of 5 mL using a 3 kDa cutoff concentrator. The concentrated DARPin D1 solution was applied to a Superdex 75 16/60 column for size-exclusion chromatography and eluted with buffer containing 20 mM Tris-HCl pH 7.5, 150 mM NaCl, and 2 mM DTT. Respective fractions were pooled together, concentrated to a final DARPin D1 concentration of ~25 mg/mL using a 3 kDa cutoff concentrator, flash-frozen in liquid nitrogen in 50 µL aliquots, and stored at -80 °C. The concentration was determined in mg/mL using a Nanodrop-1000 instrument against the size exclusion buffer using an extinction coefficient of 0.416 $Lg^{-1}cm^{-1}$.

A sequence of solution-based tubulin polymerization and depolymerization steps was performed to extract active and non-aggregated tubulin from the lyophilized tubulin stocks. On ice, the lyophilized tubulin is dissolved and diluted to 10 mg/ml with a polymerization buffer containing 50 mM MES-KOH pH 6.8, 0.5 mM EGTA, 0.4 mM GTP, 6 mM $MgCl_2$, and 33% glycerol. The lyophilized pellet was dissolved over 30 minutes by regular shaking and pipette resuspension on ice. Afterward, the clear solution was ultracentrifuged at 150,000 × g at 4 °C for 10 minutes. The supernatant was carefully

extracted, transferred to pre-warmed tubes, and incubated for 25 minutes in a water-filled heating block at 37 °C. Successful tubulin polymerization is indicated by a change of solution`s turbidity and viscosity. The solution was ultracentrifuged at 300,000 × g at 30 °C for 15 minutes and the supernatant carefully discarded. The remaining tubulin pellet was poured over with depolymerization buffer containing 80 mM Pipes-KOH pH 6.8, 0.5 mM EGTA, 2 mM GDP, 1 mM $MgCl_2$, and 2 mM $CaCl_2$. After 15 minutes of incubation, the pellet was resuspended with a pipette, and the solution was incubated again on ice for an additional 15 minutes. This step was repeated until the pellet was dissolved entirely. The depolymerized tubulin solution was diluted with depolymerization buffer to a concentration of 9.8 mg/ml and stored at −80 °C.

For crystallization, the tubulin-DARPin D1 (TD1) protein complex was formed by mixing the respective components in a 1:1.1 molar ratio. The TD1 complex was crystallized using EasyXtal 15-well plates by the hanging drop vapor diffusion method (drop size 2 µl, drop ratio 1:1, 8 drops per well) at a concentration of 9.8 mg/ml at 20 °C with a precipitant solution containing 21% (w/v) PEG 3000, 0.2 M ammonium sulfate, and 0.1 M bis-tris methane, pH 5.5. All drops were subsequently hair-seeded with crystalline material obtained in previous crystallization trials to increase the homogeneity and density of crystals. After 48 hours, crystals were washed off the plates with precipitant solution, collected in 0.6 ml tubes, and vortexed for a few seconds. This procedure induces batch crystallization within the tubes and a sedimented crystal pellet was formed after 24 hours of incubation at 20 °C. Initially, crystals grew as long needles that were broken into smaller fragments of approximately 20 × 20 × 5 μm^3 during the sample preparation.

Concentrated crystals were incubated with the non-binding trans-SBTubA4 at a concentration of 2.5 mM and then illuminated for 10 minutes at 385 nm to induce majority ligand isomerization towards the binding cis-isomer. For both measured TD1 samples (the SBTubA4 incubated, and the compound-free apo protein state), incorporation into HEC was carried out using a 3-way coupler [4]. In the process, crystals were crushed to a final size of 20 × 5 × 5 µm. The respective crystal slurry was gently mixed in a 1:1 volumetric ratio with a 22% (w/v) Hydroxyethylcelluose matrix prepared in water.

Sample preparation: tubulin T_2R-TTL protein complex

Bovine brain tubulin was purchased from the Centro de Investigaciones Biológicas (Microtubule Stabilizing Agents Group), CSIC, Madrid, Spain. Detailed descriptions and protocols of how to produce necessary proteins and how to assemble the complex are presented in ref. [2].

The plasmid encoding for the rat stathmin-like domain of *Rattus norvegicus* RB3 in a pET3d vector is described in ref. [4,5]. On ice, 0.5 µl of the plasmid was added to chemically competent *E.coli* BL21 (DE3) cells and incubated for 10 minutes. The cells were heat-shocked at 42 °C for 15 seconds and incubated for 10 minutes on ice. 100 µL of SOC medium were added and the cells incubated for 2 hours at 37 °C applying constant shaking at 180 rpm. The cells were plated on an ampicillin- supplemented LB agar plate and incubated overnight at 37 °C. The next day, 2 mL of pre-warmed LB medium was given on top of the incubated plate and the cells were loosened from the plate surface by resuspending with a pipette. The suspension was then transferred to 200 mL of pre-warmed LB medium supplemented with 200 µL of ampicillin stock solution (100 mg/mL). After the cells were incubated for 1 hour at 37 °C applying constant shaking at 180 rpm, 10 ml of cell solution were added to 1 L of pre-warmed LB medium supplemented with 1 mL of ampicillin solution (100 mg/mL) in 2 L Erlenmeyer flask and incubated at 37 °C at 180 rpm. When the optical density $OD_{600\ nm}$ reached a value of 0.7, protein expression was induced by adding 1 mL of IPTG solution (1 M). The cultures were incubated for 4 hours at 37 °C under constant shaking at 180 rpm. Finally, the cells were harvested by centrifugation at 5,000 x g at 4 °C for 30 minutes. The supernatant was removed, and the cell pellets were combined in 50 mL Falcon tubes, flash-frozen in liquid nitrogen, and stored at -80 °C. For RB3 protein purification, the frozen cell pellet (40-50 g) was first thawed in a water bath at 25 °C and then resuspended with 50 mL of lysis buffer (20 mM Tris-HCl pH 8.0, 1 mM EGTA, 2 mM DTT, DNAse I (25 µg/mL) and a protease inhibitor cocktail tablet (Roche). The cell walls were lysed on ice using an ultrasonic cell disrupter applying alterations of 2 seconds on and off pulses with 30% amplitude for 22 minutes. The lysate was centrifuged at 20,000 x g at 4 °C for 40 minutes, and the supernatant collected in a 50 mL Falcon tube. The Falcon tube was placed in boiling water for 30 minutes. The lysate thereby turns white and turbid. The lysate was centrifuged again for 40 minutes at 4 °C and 20,000 x g. The supernatant was filtered using a 0.45 µm pore size and applied to a Q Sepharose column on a FPLC system. The column was equilibrated and washed with 20 column volumes of RB3 anion-binding buffer (20 mM Tris-HCl pH 8.0, 1 mM EGTA, 2 mM DTT). The RB3 protein was eluted over 20 column volumes with a linear gradient

to 50% RB3 anion-elution buffer (20 mM Tris-HCl pH 8.0, 1 M NaCl, 1 mM EGTA, 2 mM DTT). Presence of the RB3 protein in the eluted fractions was verified by SDS-PAGE. Identified fractions were pooled together and concentrated to 5 mL volume using a 3 kDa cut-off concentrator. The concentrated solution was then applied to a Superdex 75 16/60 column and eluted with RB3 SEC buffer (10 mM HEPES pH 7.2, 150 mM NaCl, and 2 mM DTT). The protein fractions identified by absorption at 280 nm were pooled together and concentrated to 20 mg/mL RB3 using a 3 kDa cut-off concentrator and flash frozen in 30 µL aliquots of RB3 and stored at -80 °C.

The gene encoding chicken TTL was amplified from a *Gallus gallus* whole brain cDNA library (BioChain), and transferred into a negative selection vector NSKn1 containing a C-terminal hexahistidine-tag. See ref. [6]. for details. On ice, 0.5 µL of the plasmid was added to chemically competent *E.coli* BL21 (DE3) cells and incubated for 10 minutes. The cells were heat-shocked at 42 °C for 15 seconds and incubated for 10 minutes on ice. 100 µL of SOC medium were added and the cells incubated for 2 hours at 37 °C applying constant shaking at 180 rpm. The cells were plated on a kanamycin-supplemented LB agar plate and incubated overnight at 37 °C. The next day, 2 mL of pre-warmed LB medium was given on top of the incubated plate and the cells loosen from the plate surface by resuspending with a pipette. The suspension was transferred to 200 mL of pre-warmed LB medium supplemented with 200 µL of kanamycin stock solution (50 mg/mL). After the cells were incubated for 1 hour at 37 °C and 180 rpm shaking, 10 ml of cell solution were added to 1 L of pre-warmed LB medium supplemented with 1 mL of kanamycin solution (50 mg/mL) in 2 L Erlenmeyer flask and incubated at 37 °C at 180 rpm. When the optical density OD_{600nm} reached a value of 0.7, the flask were transferred to a different incubator and cooled down to 18 °C for 30 minutes. The protein expression was then induced by adding 1 mL of IPTG solution (1 M) to the Erlenmeyer flasks. The cultures were incubated overnight at 18 °C under constant shaking at 180 rpm. The cells were harvested by centrifugation at 5,000 x g at 4 °C for 30 minutes. The supernatant was removed, and the cell pellets were combined in 50 mL Falcon tubes, flash-frozen in liquid nitrogen, and stored at -80 °C. For protein purification, a frozen cell pellet (40-50 g) was first thawed in a water bath at 25 °C and then resuspended with 50 mL of lysis buffer (50 mM Tris-HCl pH 7.5, 500 mM NaCl, 20 mM imidazole, 10% glycerol, 5 mM β-mercaptoethanol, DNAse I (25 µg/mL) and a protease inhibitor cocktail tablets (Roche). The cell walls were lysed on ice using an ultrasonic cell disrupter applying alternations of 2 seconds on and off pulses with 30% amplitude for 22 minutes. The lysate was centrifuged at 20,000 x g at 4 °C for 40 minutes, and the supernatant was collected. The supernatant was filtered using a 0.45 µm pore size and applied to a Ni-NTA column on a fast protein liquid chromatography system (FPLC). After loading, the column was washed first with 30 column volumes of IMAC-binding buffer (50 mM Tris-HCl pH 7.5, 500 mM NaCl, 20 mM imidazole, 10% glycerol, 5 mM β-mercaptoethanol) and followed by additional 20 column volumes of IMAC-binding buffer combined with 4% IMAC-elution buffer (50 mM Tris-HCl pH 7.5, 250 mM NaCl, 500 mM imidazole, 10% glycerol, 5 mM β- mercaptoethanol). Protein elution was performed over 10 column volumes with 100% IMAC-elution buffer. The presence of TTL protein was confirmed by analyzing the eluted fractions on SDS-PAGE. All fractions were collected and concentrated to a final volume of 5 mL using a 10 kDa cutoff concentrator. The concentrated TTL protein solution was applied on a Superdex 200 16/60 column for size-exclusion chromatography and eluted with buffer containing 50 mM Bis-tris propane pH 7.5, 200 mM NaCl, 2.5 mM $MgCl_2$, 1% glycerol, and 2 mM DTT. Fractions were identified by absorption at 280 nm and were pooled together and concentrated to a final TTL protein concentration of 20 mg/mL using a 10 kDa cutoff concentrator, and flash-frozen in liquid nitrogen in 30 µL aliquots and stored at -80 °C.

The tubulin T_2R-TTL protein complex assembly directly follows up on the mammalian tubulin preparation described above for the Tubulin DARPin D1 (TD1) protein complex. The prepared tubulin is applied to a desalting PD-10 column at 4 °C to exchange and desalt the buffer system. The tubulin is eluted with a desalting buffer containing PIPES-KOH pH 6.8, 0.3 mM $MgCl_2$, 0.2 mM EGTA, and 0.1 mM GDP in 0.5 mL fractions. The fractions containing the tubulin are determined using a UV/Vis spectrophotometer and pooled together. The tubulin concentration was then measured against the desalting buffer using a Nanodrop-1000 instrument applying an extinction coefficient ε = 115,000 M^{-1} cm^{-1} to obtain the molar concentration, and 1.13 Lg^{-1} cm^{-1} to obtain the concentration in mg/mL. According to the determined tubulin concentration, a sixfold molar excess of RB3 is added while working on ice. The mixture was gently mixed by pipetting. Afterwards, a sixfold molar excess of TTL is added and the mixture again gently mixed by pipetting. The T_2R-TTL protein complex was concentrated to a molar equivalent of 200 µM at 4 °C using a 10 kDa cut-off concentrator. Finally, according to the resulting volume, DTT is added to a final concentration of 10 mM, both GDP and AppCp are added to

a final concentration of 1 mM each. The complex was aliquoted in 30 µL volumes, flash-frozen in liquid nitrogen and stored at -80 °C.

For the large-scale protein crystal production essential for serial crystallography experiments at XFELs, the T_2R-TTL tubulin complex was concentrated to 20 mg/mL. In PCR tubes, first 4 µL of protein complex solution was provided and then carefully mixed with additional 6 µL of precipitant solution containing 6% (w/v) PEG 4000, 30 mM $MgCl_2$, 30 mM $CaCl_2$, 100 mM MES/Imidazole, and 5 mM L-tyrosine. The PCR tubes were sealed with Parafilm and stored for two days at room temperature. The formed pellet of crystals with a homogenous size distribution of 30 × 5 × 5 µm was carefully resuspended and several setups were combined in a 1.5 mL Eppendorf tube. After the crystals have settled again, the supernatant was removed and the crystals used for compound incubation without any further concentration.

For the single compound structures, concentrated crystals were incubated for 45 minutes with 1 mM of respective commercially available approved anti-tubulin drugs, namely colchicine, epothilone A, vinblastine, and ansamitocin P3. For the photochemical affinity switch SolQ2Br, 4 mM of inactive trans isomer were added and the concentrated crystals then illuminated for 45 minutes at 385 nm to drive majority isomerisation to the active cis-isomer. For the cocktail structure, 1mM each of colchicine, epothilone A, vinblastine, peloruside A, and ansamitocin P3 were added to the concentrated crystal solution. The crystals were then immediately prepared for injection into the X-ray beam. For all described T_2R-TTL crystal systems, 20 µL of concentrated crystals were homogenously and gently embedded into 20 µL of a 22% (w/v) Hydroxyethylcelluose matrix using Hamilton syringes and a 3-way-coupler [4].

[1] Pecqueur, L. *et al.* A designed ankyrin repeat protein selected to bind to tubulin caps the microtubule plus end. *Proc. Natl. Acad. Sci. U. S. A.* **109**, 12011–12016 (2012)

[2] Tobias Muhlethaler *et al.* Crystallization Systems for the High-Resolution Structural Analysis of Tubulin–Ligand Complexes. *Methods Mol. Biol.* **2430**, 349 (2022)

[3] Wranik, M. et al. Watching the release of a photo- pharmacological drug from tubulin using time-resolved serial crystallography. Nat. Commun. **14**, 903 (2023)

[4] James, D. *et al.* Improving High Viscosity Extrusion of Microcrystals for Time-resolved Serial Femtosecond Crystallography at X-ray Lasers. *J. Vis. Exp.* e59087 (2019)

[5] Ozon S., Maucuer A., Sobel A. The stath-min family – molecular and biological charac- terization of novel mammalian proteins expressed in the nervous system. Eur J Bio-chem **248**, 3 (1997)

[6] Ravelli R. B., Gigant B., Curmi P.A., Jourdain I., Lachkar S., Sobel A., Knossow M. Insight into tubulin regulation from a complex with colchicine and a stathmin-like domain. Nature **428**, 6979 (2004)

[7] Olieric, N. *et al.* Automated seamless DNA co-transformation cloning with direct expression vectors applying positive or negative insert selection. *BMC Biotechnol* **10**, 56 (2010)

Crystallographic Table 1: Data processing Statistics

	**Lysozyme (LCP)**	**Lysozyme (LCP) aniso**	**Lysozyme (HEC)**	**Lysozyme (HEC) aniso**	**KR2 (dark)**	**KR2 (dark) aniso**	**KR2 (light)**	**KR2 (light) aniso**	**TD1 (apo)**	**TD1 (apo) aniso**	**TD1 (SBTubA4)**	**TD1 (SBTubA4) aniso**	**T_2R-TTL (apo)**	**T_2R-TTL (SolQ2 Br)**	**T_2R-TTL (Colchi-cine)**	**T_2R-TTL (Epothi-lone A)**	**T_2R-TTL (Vinblas-tine)**	**T_2R-TTL (Ansami-tocin P3)**	**T_2R-TTL (Cocktail)**
**Space group**	$P4_32_12$				I222				$P2_1$				$P2_12_12_1$						
a, *b*, *c* (Å)	77.9, 77.94, 37.64,		77.9, 77.9, 37.69		41.46, 84.49, 234.95				73.47, 91.19, 82.70				106.53, 160.48, 181.24						
a, b, g (°)	90, 90, 90		90, 90, 90		90, 90, 90				90, 96.43, 90				90, 90, 90						
**Indexed patterns**	30993		25690		53745		25036		13466		48475		15862	22587	11036	10258	1939	2022	22402
**Indexing rate (%)**	50		50		6.8		11.7		9.1		31.3		21.7	14.1	10.5	10.8	6.5	5.7	10.0
**Resolution range for overall and high-resolution statistics**	14.995 – 1.31 (1.36 – 1.31)	14.995 – 1.31 (1.36 – 1.31)	14.968 – 1.32 (1.37 – 1.32)	14.968 – 1.32 (1.37 – 1.32)	15.976 – 1.98 (2.06 – 1.98)	15.976 – 1.76 (1.83 – 1.76)	15.10 – 2.04 (2.11 – 2.04)	15.10 – 1.76 (1.83 – 1.76)	14.94 - 2.30 (2.38 - 2.30)	14.94 - 2.10 (2.18 - 2.10)	14.96 - 2.00 (2.10 - 2.00)	14.96 - 1.80 (1.90 - 1.80)	15.22 – 2.40 (2.50 – 2.40)	15.35 – 2.60 (2.70 – 2.60)	15.81 – 2.70 (2.80 – 2.70)	15.26 – 2.60 (2.70 – 2.60)	15.13 – 3.10 (3.20 – 3.10)	15.17 – 3.10 (3.20 – 3.10)	15.29 – 2.40 (2.50 – 2.40)
**No. reflections**	15115 (1466)	13352 (167)	14807 (1436)	13094 (68)	30862 (3034)	28894 (370)	28612 (2818)	26536 (172)	48103 (4733)	40528 (761)	72367 (7184)	73468 (1532)	121578 (12016)	95765 (9439)	85408 (8462)	95755 (9439)	56577 (5570)	56426 (5544)	121575 (12016)
**Completeness (%)**	99.85 (98.59)	89.39 (11.23)	100 (100)	88.43 (4.74)	100 (100)	65.7 (8.56)	100 (100)	60.4 (3.98)	100 (100)	64.2 (12.1)	100 (100)	74.1 (15.5)	100 (100)	100 (100)	100 (100)	100 (100)	100 (100)	100 (100)	100 (100)
**Multiplicity**	423.46 (12.6)	471.23 (17.6)	739.53 (24.2)	832.82 (49.1)	939.91 (479.0)	960.89 (234.7)	390 (224.5)	400.15 (115.0)	79.9 (49.3)	88.4 (79.1)	210.7 (147.5)	217.2 (216.8)	185.5 (98.7)	381.3 (251.9)	138.4 (90.8)	209.7 (137.4)	42.8 (31.9)	38.5 (28.7)	350.9 (177.6)
**R_{split} (%)**	11.93 (155.46)	11.74 (104.39)	11.16 (163.54)	10.86 (113.61)	9.36 (98.87)	9.16 (78.52)	13.59 (100.07)	13.30 (93.33)	23.0 (102.2)	21.8 (52.0)	16.9 (58.8)	16.8 (54.4)	20.6 (194.2)	15.2 (183.1)	22.6 (168.9)	21.8 (119.3)	39.6 (129.8)	36.6 (94.3)	14.1 (139.6)
**$CC_{1/2}$**	0.975 (0.204)	0.947 (0.304)	0.996 (0.196)	0.982 (0.473)	0.986 (0.689)	0.986 (0.483)	0.967 (0.632)	0.966 (0.315)	0.929 (0.612)	0.972 (0.677)	0.964 (0.766)	0.964 (0.670)	0.951 (0.190)	0.976 (0.257)	0.944 (0.207)	0.983 (0.370)	0.771 (0.26)	0.776 (0.420)	0.980 (0.321)
CC*	0.993 (0.582)	0.993 (0.683)	0.995 (0.573)	0.995 (0.801)	0.996 (0.904)	0.996 (0.542)	0.991 (0.879)	0.991 (0.439)	0.982 (0.871)	0.981 (0.898)	0.991 (0.931)	0.991 (0.895)	0.987 (0.460)	0.994 (0.640)	0.985 (0.586)	0.983 (0.735)	0.925 (0.642)	0.935 (0.770)	0.995 (0.697)
**<*I/σ(I)*>**	7.31 (0.17)	8.14 (0.99)	7.29 (0.68)	8.16 (0.89)	7.57 (1.10)	8.17 (1.46)	5.69 (1.08)	6.18 (1.23)	4.51 (0.92)	5.29 (1.71)	6.11 (1.59)	6.13 (1.58)	3.88 (0.41)	6.13 (0.59)	4.56 (0.64)	4.72 (0.89)	3.07 (0.90)	3.38 (1.25)	6.15 (0.77)
**PDB Code**	8CL5		8CL6		8CL7		8CL8		8CL9		8CLA		8CLC	8CLF	8CLB	8CLG	8CLE	8CLD	8CLH

Crystallographic Table 2: Data Refinement Statistics

	**Lysozyme (LCP)**	**Lysozyme (HEC)**	**KR2 (dark)**	**KR2 (light)**	**TD1 (apo)**	**TD1 (SBTubA4)**	**T_2R-TTL (apo)**	**T_2R-TTL (SolQ2 Br)**	**T_2R-TTL (Colchicine)**	**T_2R-TTL (Epothilone A)**	**T_2R-TTL (Vinblastine)**	**T_2R-TTL (Ansamitocin P3)**	**T_2R-TTL (Cocktail)**
**Resolution (Å)**	14.47 – 1.31	13.54 – 1.32	15.97 – 1.76	14.83 – 2.40	13.00 – 2.50	13.00 – 2.00	15.20 – 2.70	15.35 – 2.70	15.18 – 3.00	15.26 – 2.80	15.13 – 3.20	15.10 – 3.20	15.29 – 2.50
**No. reflections**	26527	25665	28709	15718	34519	68170	85293	85186	62163	76658	51318	50716	108000
**R_{work} / R_{free} in %**	12.46 / 14.79	12.08 / 15.58	15.22 / 18.24	33.21/ 36.03	18.79 / 24.13	15.60 / 20.58	20.02 / 25.05	18.68 / 23.44	17.37 / 23.02	17.91 / 22.67	20.07 / 24.79	20.05 / 24.12	18.54 / 23.90
**No. atoms**	1329	1343	2491	2394	8135	8457	17587	17801	17723	18117	17609	17506	17984
**Protein**	1199	1190	2167	2095	7925	7945	17331	17471	17349	17817	17393	17294	17456
**Ligands**	16	24	214	236	65	54	56	56	56	56	56	28	56
**Water**	114	129	110	63	145	424	91	46	37	44	33	4	126
**B-factor**	24.17	24.08	39.51	39.65	37.43	32.47	78.29	80.46	68.31	67.55	74.44	64.38	78.83
**Protein**	21.69	21.08	36.08	38.36	37.62	32.33	78.50	80.63	68.46	67.59	74.72	64.40	78.90
**Ligands**	51.54	53.37	68.47	51.71	29.62	34.81	66.52	69.28	60.50	49.35	61.15	39.11	65.54
Water	46.36	46.28	51.26	37.27	30.44	36.34	51.99	54.59	46.98	45.75	57.62	30.57	52.94
Bond length (Å)	0.016	0.014	0.010	0.007	0.002	0.014	0.002	0.004	0.001	0.009	0.006	0.003	0.004
***Bond angles (*°)**	1.421	1.503	1.116	0.934	0.460	1.448	0.462	0.657	0.401	1.353	0.995	0.700	1.087
**Ramachandran favored / allowed / outliers in %**	99.21 / 0.79 / 0.00	98.43 / 1.57 / 0.00	97.72 / 2.28 / 0.00	96.56 / 3.44/ 0.00	96.33/ 3.47 / 0.20	96.26 / 3.35 / 0.39	96.25 / 3.71 / 0.05	96.25 / 3.71 / 0.05	96.20 / 3.71 / 0.09	95.99 / 3.56 / 0.46	96.10 / 3.34 / 0.56	97.59 / 2.36 / 0.05	96.54 / 3.28 / 0.18
**PDB Code**	8CL5	8CL6	8CL7	8CL8	8CL9	8CLA	8CLC	8CLF	8CLB	8CLG	8CLE	8CLD	8CLH

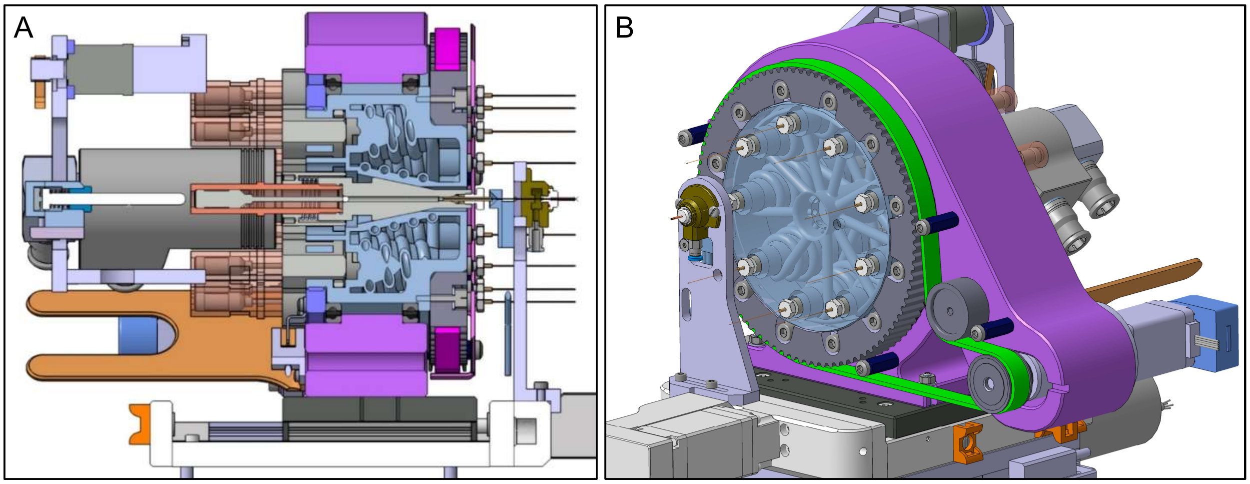

Supplementary Figure 1. Liquid feedthrough channels of the reservoir holder drum. Panel A shows a vertical cut through of the drum and a reservoir assembly. Panel B shows an oblique view device with the reservoir holder drum in a transparent material so that the arrangement of the liquid feedthrough channels is visible within the 3D printed steel.

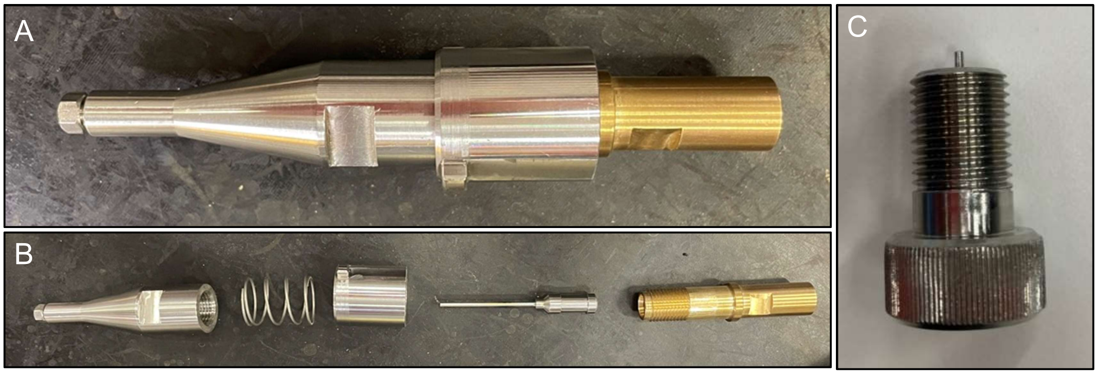

Supplementary Figure 2. The reservoir assembly and tool for Teflon ball insertion.Panel A shows the assembled reservoir assembly body and panel B contains the components of the reservoir assembly in an exploded view. The tool made in house for reliable insertion of the Teflon ball within the reservoir channel. The made-in-house tool, shown in panel C, is used for reliable insertion of the Teflon ball which is sealing the sample within the reservoir channel.

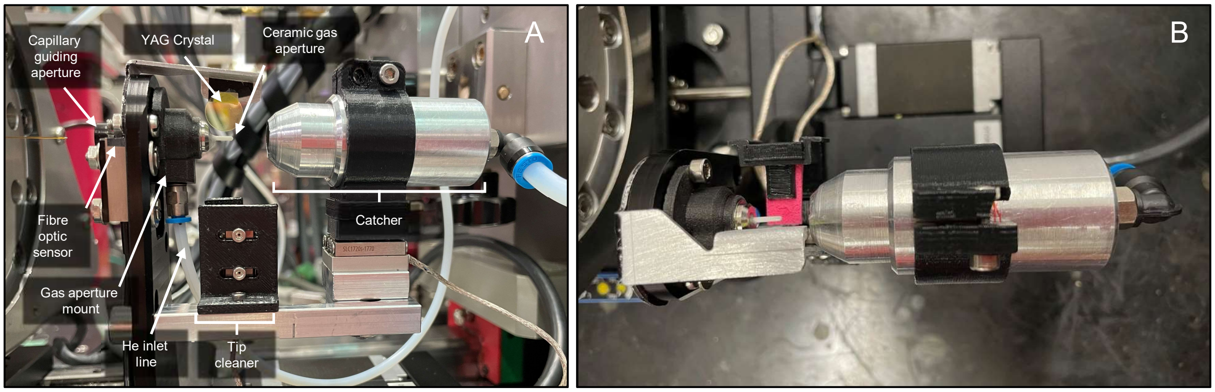

Supplementary Figure 3. The sample interaction region. Panel A shows a side on view with all features annotated. In this panel the arm of the tip cleaner is removed so that other elements are clearly visible. Panel B shows a top view with the arm of the tip cleaner in place with the pink sponge clearly visible.

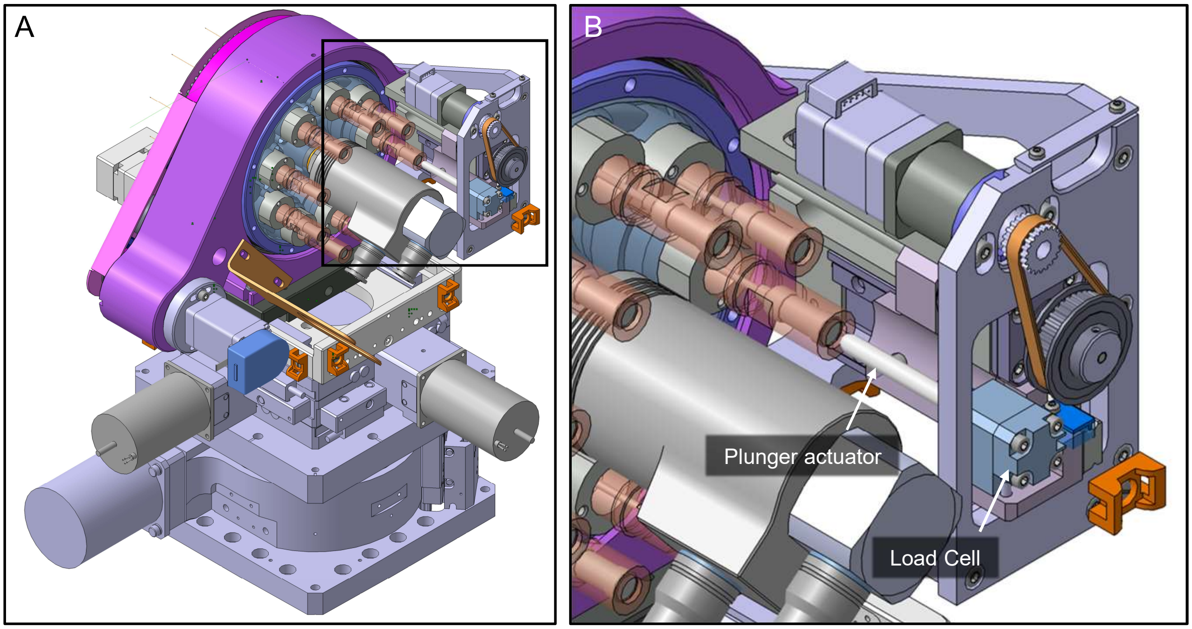

Supplementary Figure 4. Detailed view of the plunger assembly. Panel A shows the plunger assembly with inset square magnified in panel B. The plunger actuator, which contacts the plunger within the reservoir assembly and controls extrusion is indicated. The load cell registers the force exerted on the plunger rod and provides a measure of pressure exerted on the plunger rod.

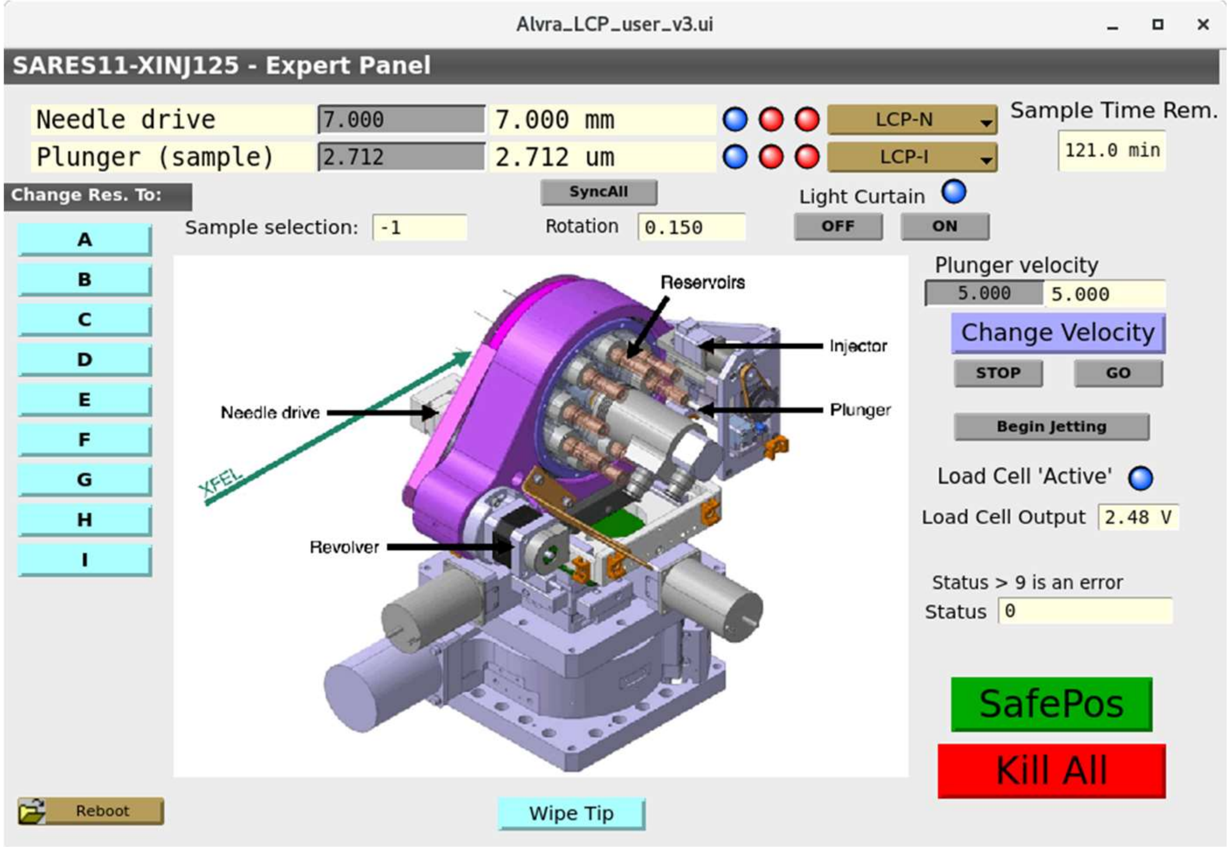

Supplementary Figure 5. Graphical user interface for the multi-reservoir high viscosity extruder. Reservoir positions A-H can be chosen with the blue buttons on the left of the panel – these initiate the automatic loading sequence of the chosen reservoir. The light curtain used for positional feedback of the capillary tip during the loading sequence can be toggled off once the reservoir assembly is in place in order to avoid light contamination of the sample. The ‘Load Cell Output’ readback value represents the force exerted on the plunger actuator as a voltage – the ‘Load Cell ‘Active’’ indicator turns blue when the plunger is in contact with the sample and grey in other instances. The desired plunger velocity in μm/s is typed in by the user and then jetting is initiated by clicking the ‘Begin Jetting’ button. The ‘Wipe Tip’ button triggers an automated sequence to clean debris from the tip of the capillary needle using a sponge.

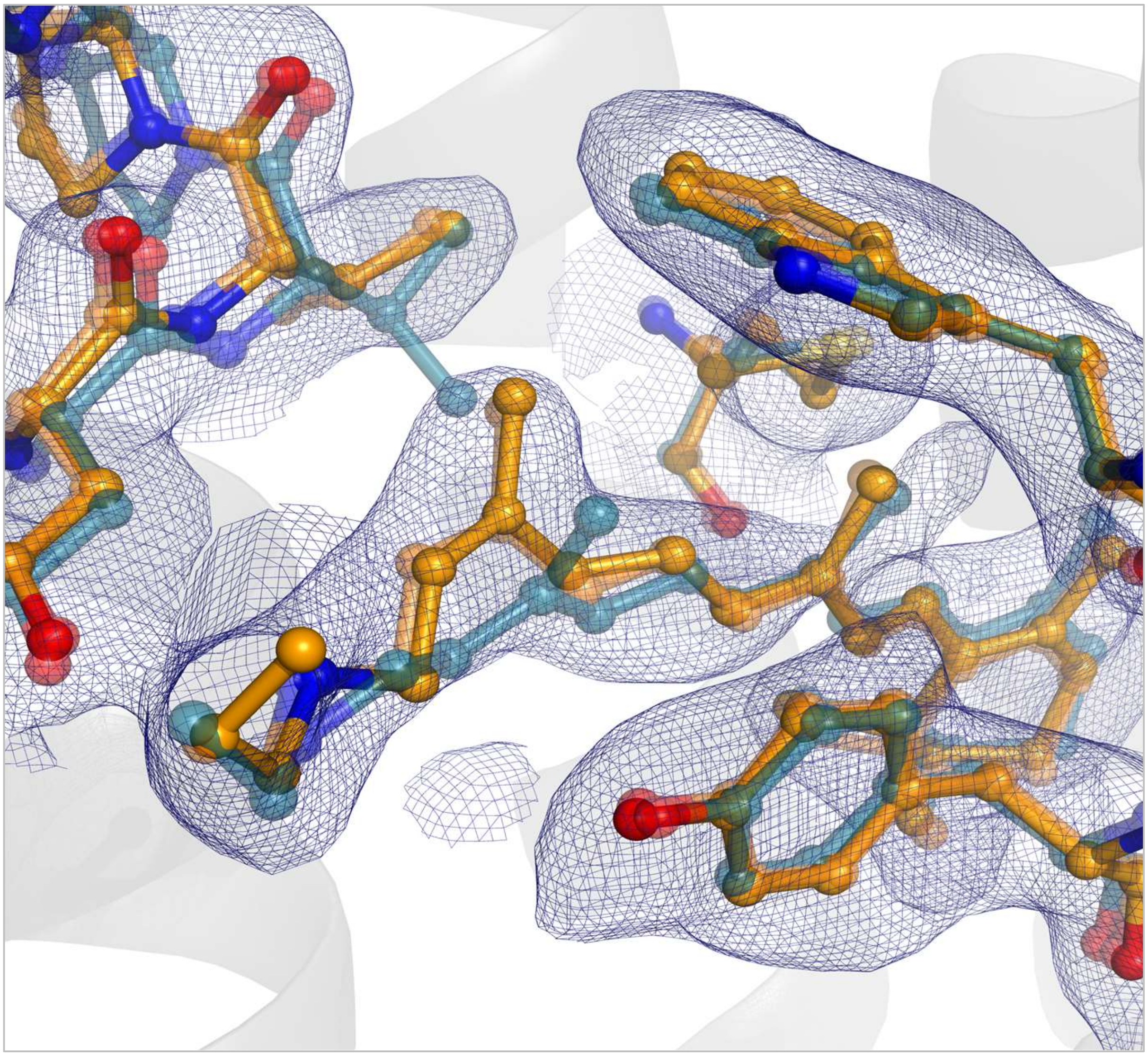

Supplementary Figure 6. KR2 extrapolated map, $2F_{ext}–F_c$ at 19% activation level for 1 μs time delay presented around the retinal pocket. Yellow model depicts the refined KR2 structure in extrapolated map and blue and orange transparent models represent dark 6TK6 and 1 ms light 6TK2 structures. Electron density shown at sigma level of 1.0.

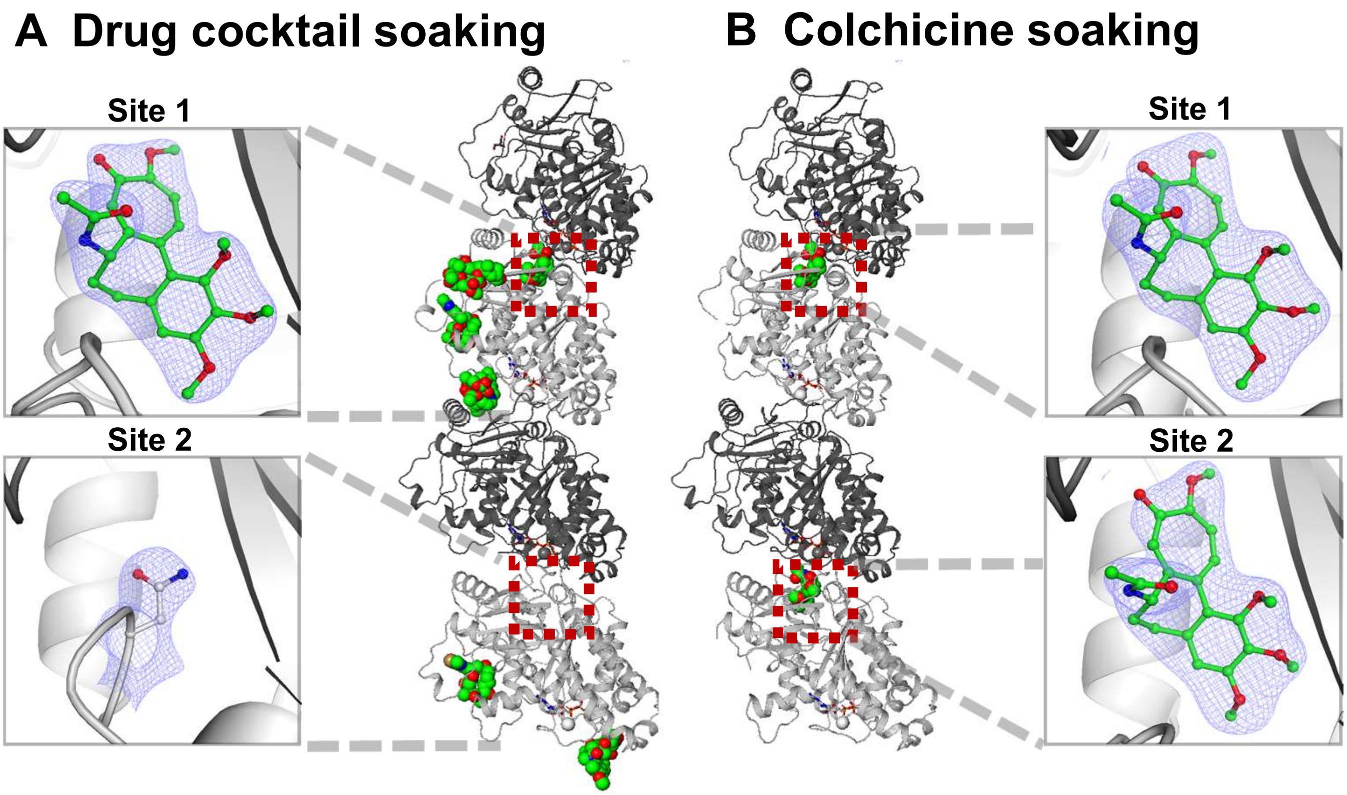

Supplementary Figure 7. Global effects of multiple ligand binding. (A) Cocktail of ligands facilitates the exploration of global effects whereby simultaneous ligand mixing might influence affinity levels at different binding sites as compared to (B) mixing with a single compound. For both soaks identical concentration of Colchicine ligand were used. Electron denisty ($2F_o$-F_c) maps are shown at sigma level of 1.0. The apo protein (A, site 2) is indicated by the flipped-in residue ASN247.

Site 1 – high occupancy binding

Standard omit maps

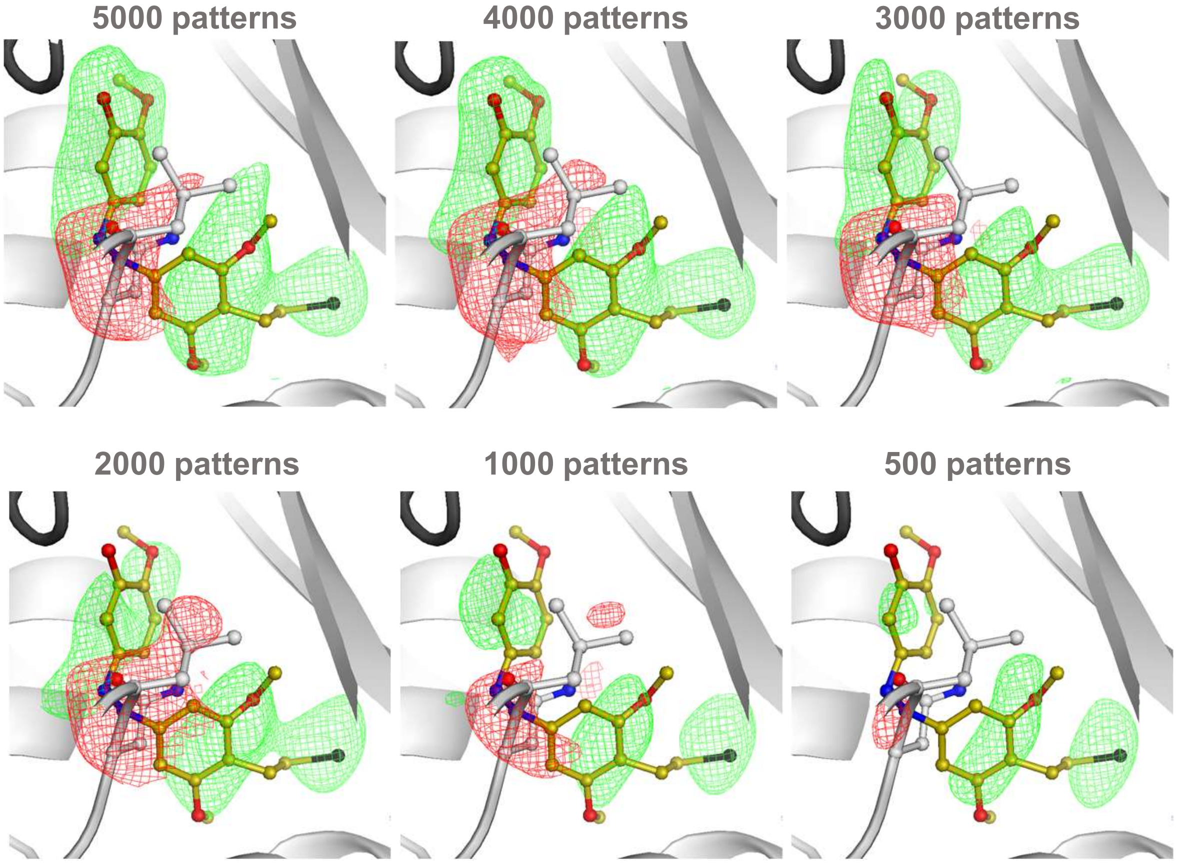

Isomorphous difference density maps

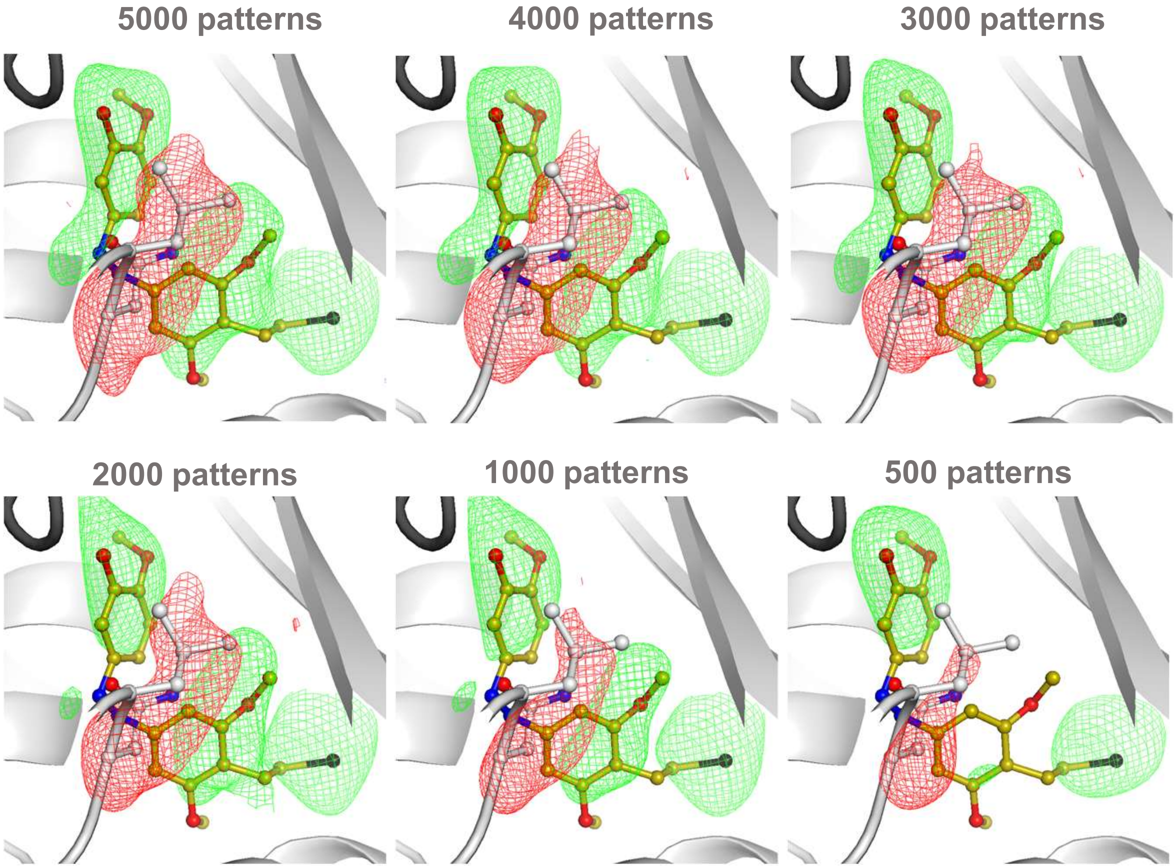

Supplementary Figure 8. Titration series of minimal amount of data required to reliably detect SolQ2Br binding within the high occupancy binding site 1. Comparison between standard omit (F_o-F_c) maps against apo protein state and isomorphous difference density (F_o-F_c) maps. The SolQ2Br structure is overlaid for better illustration. All maps are shown at a sigma level of 1.0. Positive difference density is shown in green, negative difference density in red.

Site 2 – low occupancy binding

Standard omit maps

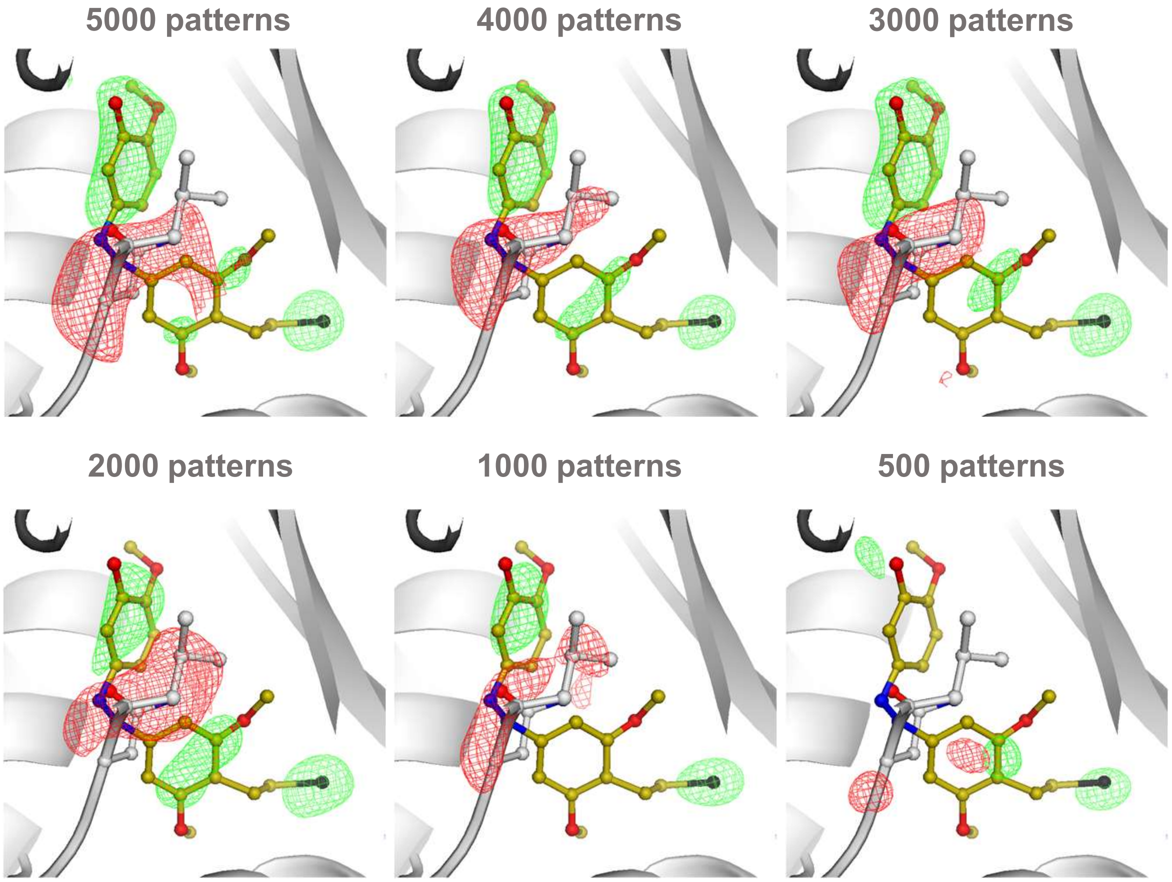

Isomorphous difference density maps

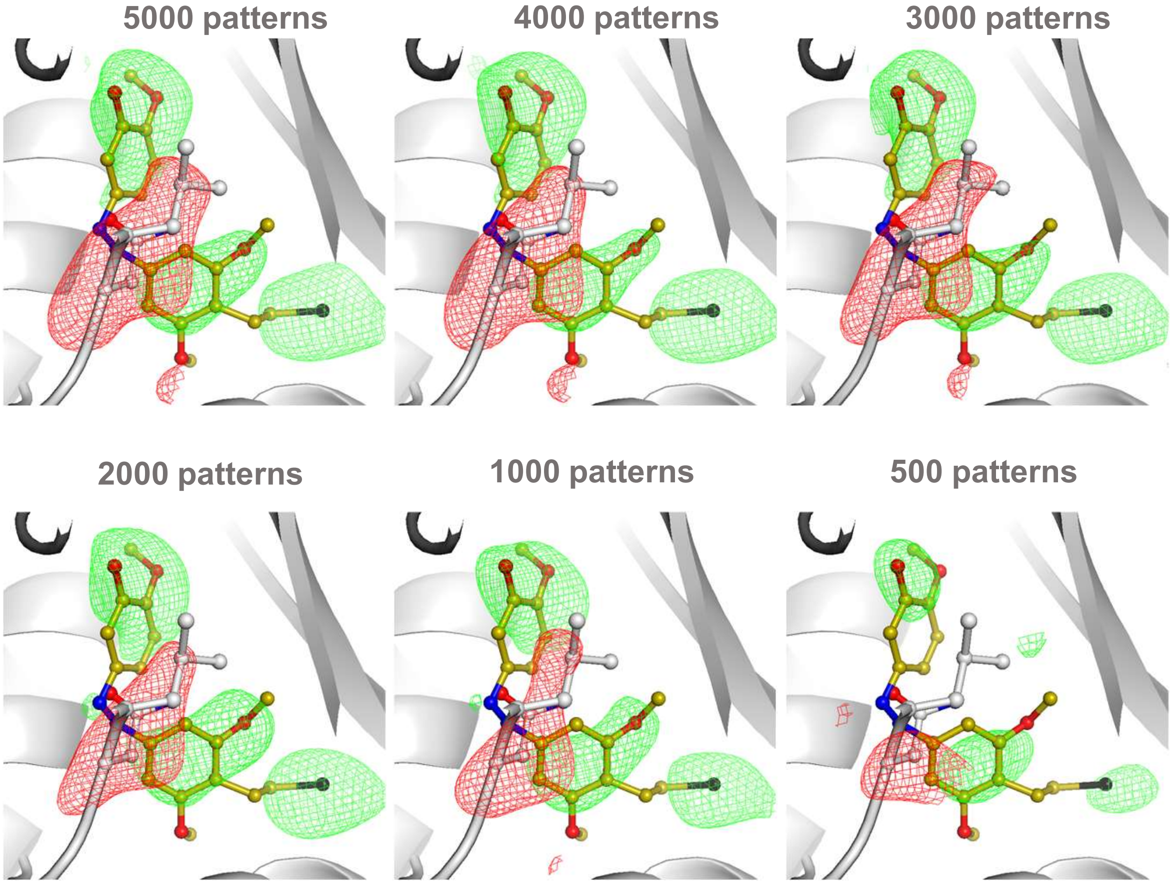

Supplementary Figure 9. Supplementary figure S8. Titration series of minimal amount of data required to reliably detect SolQ2Br binding within the low occupancy binding site 2. Comparison between standard omit (F_o-F_c) maps against apo protein state and isomorphous difference density (F_o-F_c) maps. The SolQ2Br structure is overlaid for better illustration. All maps are shown at a sigma level of 1.0. Positive difference density is shown in green, negative difference density in red.

Plunger Speed (µm/s)	Measured speed (µm/s)	Calculated Speed (µm/s)	Ratio calulcated/actual
1	916	860	93.8%
2	1619	1721	106.3%
5	4647	4302	92.6%
10	8439	8604	102.0%
15	14568	12907	88.6%
20	18979	17208	90.7%
25	26490	21511	81.2%
30	30960	25813	83.4%

Supplementary Table 1: Sample extrusion speeds obtained from fast camera recordings. The calculated speed was estimated by solving volumetric flow rate equation.
