# Supplementary material for: A multi-reservoir extruder for time-resolved serial protein crystallography and compound screening at X-ray free-electron lasers
Source: Nat Commun. 2023 Dec 2;14:7956. doi: 10.1038/s41467-023-43523-5 (PMC10693631; doi:10.1038/s41467-023-43523-5)
Supplement: Supplementary file 3 — Description of Additional Supplementary Files [file 41467_2023_43523_MOESM3_ESM.pdf]

Description of Additional Supplementary Files

File Name: Supplementary Movie 1
Description: Movie depicting the multi-reservoir extruder in operation.

File Name: Supplementary Movie 2
Description: Fast camera recording of the extrusion process.
