# Supplementary material for: A multi-reservoir extruder for time-resolved serial protein crystallography and compound screening at X-ray free-electron lasers
Source: Nat Commun. 2023 Dec 2;14:7956. doi: 10.1038/s41467-023-43523-5 (PMC10693631; doi:10.1038/s41467-023-43523-5)
Supplement: Supplementary file 6 — Reporting Summary [file 41467_2023_43523_MOESM6_ESM.pdf]

nature portfolio

Corresponding author(s): Michal Kepa

Last updated by author(s): Oct 23, 2023

Reporting Summary

Nature Portfolio wishes to improve the reproducibility of the work that we publish. This form provides structure for consistency and transparency in reporting. For further information on Nature Portfolio policies, see our Editorial Policies and the Editorial Policy Checklist.

Statistics

For all statistical analyses, confirm that the following items are present in the figure legend, table legend, main text, or Methods section.

n/a	Confirmed	
[x]	[ ]	The exact sample size (*n*) for each experimental group/condition, given as a discrete number and unit of measurement
[x]	[ ]	A statement on whether measurements were taken from distinct samples or whether the same sample was measured repeatedly
[x]	[ ]	The statistical test(s) used AND whether they are one- or two-sided<br>*Only common tests should be described solely by name; describe more complex techniques in the Methods section.*
[x]	[ ]	A description of all covariates tested
[x]	[ ]	A description of any assumptions or corrections, such as tests of normality and adjustment for multiple comparisons
[ ]	[x]	A full description of the statistical parameters including central tendency (e.g. means) or other basic estimates (e.g. regression coefficient) AND variation (e.g. standard deviation) or associated estimates of uncertainty (e.g. confidence intervals)
[x]	[ ]	For null hypothesis testing, the test statistic (e.g. *F*, *t*, *r*) with confidence intervals, effect sizes, degrees of freedom and *P* value noted<br>*Give P values as exact values whenever suitable.*
[x]	[ ]	For Bayesian analysis, information on the choice of priors and Markov chain Monte Carlo settings
[x]	[ ]	For hierarchical and complex designs, identification of the appropriate level for tests and full reporting of outcomes
[ ]	[x]	Estimates of effect sizes (e.g. Cohen's *d*, Pearson's *r*), indicating how they were calculated

Our web collection on statistics for biologists contains articles on many of the points above.

Software and code

Policy information about availability of computer code

Data collection	Data collection was performed at the Alvra endstation of Swiss X-ray Free Electron Laser and the PXI beamline of the Swiss Light Source. The use of the integrated software is available to registered users of the facilities.
Data analysis	Data was analyzed using freely available software (Crystfel 0.9.1, COOT 0.9.8.1, Phenix 1.19.2, Pymol 2.4.2) as detailed in the materials and methods section of our manuscript.

For manuscripts utilizing custom algorithms or software that are central to the research but not yet described in published literature, software must be made available to editors and reviewers. We strongly encourage code deposition in a community repository (e.g. GitHub). See the Nature Portfolio guidelines for submitting code & software for further information.

Data

Policy information about availability of data

All manuscripts must include a data availability statement. This statement should provide the following information, where applicable:

- Accession codes, unique identifiers, or web links for publicly available datasets
- A description of any restrictions on data availability
- For clinical datasets or third party data, please ensure that the statement adheres to our policy

Coordinates and structure factors have been deposited in the PDB database under accession code 8CL5 [https://doi.org/10.2210/pdb8CL5/pdb] (Lysozyme, embedded in LCP), 8CL6 [https://doi.org/10.2210/pdb8CL6/pdb] (Lysozyme, embedded in HEC), 8CL7 [https://doi.org/10.2210/pdb8CL7/pdb] (KR2, dark state), 8CL9 [https://doi.org/10.2210/pdb8CL9/pdb] (Tubulin (TD1), apo state), 8CLC [https://doi.org/10.2210/pdb8CLC/pdb] (Tubulin (T2R-TTL), apo state), 8CLF [https://

doi.org/10.2210/pdb8CLF/pdb] (Tubulin (T2R-TTL), SolQ2Br bound state), 8CLB [https://doi.org/10.2210/pdb8CLB/pdb] (Tubulin (T2R-TTL), Colchicine bound state), 8CLG [https://doi.org/10.2210/pdb8CLG/pdb] (Tubulin (T2R-TTL), Epothilone A bound state), 8CLE [https://doi.org/10.2210/pdb8CLE/pdb] (Tubulin (T2R-TTL), Vinblastine bound state), 8CLD [https://doi.org/10.2210/pdb8CLD/pdb] (Tubulin (T2R-TTL), Ansamitocin P3 bound state), 8CLH [https://doi.org/10.2210/pdb8CL/pdb] (Tubulin (T2R-TTL), drug cocktail). The model refined against extrapolated data, extrapolated structure factors, and light data used for data extrapolation have been deposited under accession codes 8CL8 [https://doi.org/10.2210/pdb8CL8/pdb] (KR2, 1µs time delay). Other data are available from the corresponding authors upon request. The source data underlying Figure 1c-d are provided as a Source Data file.

Human research participants

Policy information about studies involving human research participants and Sex and Gender in Research.

Reporting on sex and gender	No human research participants
Population characteristics	No human research participants
Recruitment	No human research participants
Ethics oversight	No human research participants

Note that full information on the approval of the study protocol must also be provided in the manuscript.

Field-specific reporting

Please select the one below that is the best fit for your research. If you are not sure, read the appropriate sections before making your selection.

☒ Life sciences ☐ Behavioural & social sciences ☐ Ecological, evolutionary & environmental sciences

For a reference copy of the document with all sections, see nature.com/documents/nr-reporting-summary-flat.pdf

Life sciences study design

All studies must disclose on these points even when the disclosure is negative.

Sample size	All statistical data on the number of measured crystals and the quality of the data is given in the supplementary crystallographic tables. The number of indexable diffraction patterns were determined using CrystFEL directly from the raw data.
Data exclusions	No data has been excluded from analysis.
Replication	Data has been collected at two separate beamtimes at Alvra, SwissFEL.
Randomization	No randomization was necessary as we did not rely on statistical analysis. As is the standard in crystallography reflections for the determination of Rfree were chosen randomly.
Blinding	No blinding was necessary as this is not a clinical study.

Reporting for specific materials, systems and methods

We require information from authors about some types of materials, experimental systems and methods used in many studies. Here, indicate whether each material, system or method listed is relevant to your study. If you are not sure if a list item applies to your research, read the appropriate section before selecting a response.

Materials & experimental systems

n/a	Involved in the study	
☒	☐	Antibodies
☒	☐	Eukaryotic cell lines
☒	☐	Palaeontology and archaeology
☒	☐	Animals and other organisms
☒	☐	Clinical data
☒	☐	Dual use research of concern

Methods

n/a	Involved in the study	
☒	☐	ChIP-seq
☒	☐	Flow cytometry
☒	☐	MRI-based neuroimaging
